# *Chlamydomonas reinhardtii* as a plant model system to study mitochondrial complex I dysfunction

**DOI:** 10.1002/pld3.200

**Published:** 2020-02-03

**Authors:** Nitya Subrahmanian, Andrew David Castonguay, Thea Aspelund Fatnes, Patrice Paul Hamel

**Affiliations:** ^1^ Department of Molecular Genetics The Ohio State University Columbus OH USA; ^2^ Plant Cellular and Molecular Biology Graduate Program The Ohio State University Columbus OH USA; ^3^ Molecular Genetics Graduate Program The Ohio State University Columbus OH USA; ^4^ Department of Biological Chemistry and Pharmacology The Ohio State University Columbus OH USA; ^5^ Present address: Fürst Medical Laboratory Oslo Norway

**Keywords:** *Chlamydomonas reinhardtii*, complex I, insertional and site‐directed mutagenesis, mitochondrial biogenesis, mitochondrial diseases

## Abstract

Mitochondrial complex I, a proton‐pumping NADH: ubiquinone oxidoreductase, is required for oxidative phosphorylation. However, the contribution of several human mutations to complex I deficiency is poorly understood. The unicellular alga *Chlamydomonas reinhardtii* was utilized to study complex I as, unlike in mammals, mutants with complete loss of the holoenzyme are viable. From a forward genetic screen for complex I‐deficient insertional mutants, six mutants exhibiting complex I deficiency with assembly defects were isolated. *Chlamydomonas* mutants isolated from our screens, lacking the subunits NDUFV2 and NDUFB10, were used to reconstruct and analyze the effect of two human mutations in these subunit‐encoding genes. The K209R substitution in NDUFV2, reported in Parkinson's disease patients, did not significantly affect the enzyme activity or assembly. The C107S substitution in the NDUFB10 subunit, reported in a case of fatal infantile cardiomyopathy, is part of a conserved C‐(X)_11_‐C motif. The cysteine substitutions, at either one or both positions, still allowed low levels of holoenzyme formation, indicating that this motif is crucial for complex I function but not strictly essential for assembly. We show that the algal mutants provide a simple and useful platform to delineate the consequences of patient mutations on complex I function.

## 1 INTRODUCTION

Mitochondrial oxidative phosphorylation (OXPHOS) involves four major membrane‐bound complexes (I, II, III, and IV) mediating electron transfer from the substrates, NADH or succinate, to the terminal electron acceptor O_2_ (Green & Tzagoloff, 1966). In concert with their oxidoreductase activities, complexes I, III, and IV also translocate protons across the mitochondrial inner membrane, thereby establishing the proton gradient necessary for complex V (F_1_F_0_ ATP synthase) to generate ATP on the matrix side (Mitchell, 1961).

With over 40 nucleus‐ and mitochondria‐encoded subunits, mitochondrial complex I is a type‐I NADH dehydrogenase (Kerscher, Dröse, Zickermann, & Brandt, 2008) and the largest respiratory complex in the mitochondrial inner membrane (Hirst, 2013). Among the ~40 subunits common to all eukaryotic complexes I (Cardol, 2011), only 14 orthologs make up the bacterial enzyme and are therefore considered to be the “core” subunits as they constitute the minimal requirement for enzymatic activity (Berrisford, Baradaran, & Sazanov, 2016). This highly conserved core is composed of seven catalytic subunits often encoded by the nuclear genome, binding the prosthetic groups (one FMN and eight iron‐sulfur (Fe‐S) clusters) required for oxidation of NADH, plus seven hydrophobic subunits (ND subunits) generally encoded in the mitochondrial genomes of eukaryotes (Remacle, Barbieri, Cardol, & Hamel, 2008). The roles of the 24 non‐core subunits conserved in all eukaryotic lineages, also referred to as “accessory subunits,” are largely unknown. It is proposed that they have supportive roles in stabilizing the complex and/or regulating its activity (Kmita & Zickermann, 2013; Stroud et al., 2016).

Complex I biogenesis in eukaryotes is a complicated process dependent upon the coordinated expression of the nuclear and mitochondrial genomes (Guerrero‐Castillo et al., 2017). This process has attracted considerable attention as ~37% of OXPHOS disorders are characterized by isolated or combined complex I deficiency (Ghezzi & Zeviani, 2018; Rodenburg, 2016). Since the first report of human complex I deficiency by Morgan‐Hughes *et al*. in 1979, pathogenic mutations have been discovered in 20 (out of 37) nuclear‐encoded subunits and all seven mitochondrially encoded subunits of complex I (Fiedorczuk & Sazanov, 2018; Friederich et al., 2017; Koopman et al., 2016; Rodenburg, 2016). These mutations have been associated with a variety of clinical symptoms including hypertrophic cardiomyopathy, Leigh syndrome, and additional neurodegenerative disorders (Koopman et al., 2016; Pagniez‐Mammeri et al., 2012; Sharma, Lu, & Bai, 2009). At the cellular level, patient‐derived fibroblasts display a diverse range of phenotypes including decreased complex I activity and assembly, increased reactive oxygen species production, mitochondrial membrane depolarization, defective ATP production, and altered mitochondrial morphology (Distelmaier et al., 2009; Giachin, Bouverot, Acajjaoui, Pantalone, & Soler‐Lopez, 2016).

Although our knowledge of complex I deficiency is broadening, the molecular mechanisms underlying the clinical symptoms remain poorly understood. There is no clear correlation between the clinical presentation and the corresponding molecular defects (Distelmaier et al., 2009). For instance, different mutations in the same gene may present with alternate clinical phenotypes. In addition, there is variability in the complex I deficiency observed at the tissue and organ levels for the same patient (Giachin et al., 2016; Shoubridge, 2001). As the heterogeneity of the biochemical and clinical phenotypes adds additional layers of complexity, demonstrating the pathogenicity of a molecular lesion in humans has become a real challenge. Therefore, some mutations have been assigned a “provisional” status because their contribution to the disease phenotype remains uncertain (Mitchell, Elson, Howell, Taylor, & Turnbull, 2006).

Due to the above‐mentioned difficulties associated with studying complex I disorders, non‐human experimental model systems have been used to dissect the molecular bases of mitochondrial complex I assembly. While bacterial systems have been previously used for reconstructing human pathogenic mutations, they lack the subunit complexity of their eukaryotic counterpart (Vinothkumar, Zhu, & Hirst, 2014). Similarly, the single‐celled eukaryote *Saccharomyces cerevisiae* is an unsuitable experimental system because it lacks mitochondrial complex I (Lasserre et al., 2015). Previously, the obligate aerobic yeasts *Yarrowia lipolytica* and *Neurospora crassa* have been successfully utilized to mimic disease‐associated mutations in genes encoding structural subunits and an assembly factor (Ahlers, Garofano, Kerscher, & Brandt, 2000; Duarte, Schulte, Ushakova, & Videira, 2005; Kerscher, Grgic, Garofano, & Brandt, 2004; Maclean, Kimonis, & Balk, 2018).

The unicellular photosynthetic alga *Chlamydomonas reinhardtii* (to be referred to as *Chlamydomonas*) has emerged as an alternative simple model system for studying mitochondrial complex I (Barbieri et al., 2011; Remacle et al., 2008; Salinas, Larosa, Cardol, Marechal‐Drouard, & Remacle, 2014). Firstly, the composition of complex I in *Chlamydomonas* is similar to its human counterpart (Cardol et al., 2004, 2008; Remacle, Hamel, Larosa, Subrahmanian, & Cardol, 2012). Secondly, the nuclear and mitochondrial genomes encoding complex I subunits are amenable to manipulation (Barbieri et al., 2011; Remacle, Cardol, Coosemans, Gaisne, & Bonnefoy, 2006). Thirdly, unlike mammalian organisms, complete loss of complex I is still viable due to the capacity of this alga to photosynthesize (Cardol et al., 2003; Massoz et al., 2015).

In addition, alternative enzymes in the *Chlamydomonas* electron transport chain (ETC) can partially bypass the lack of complex I (Lecler, Vigeolas, Emonds‐Alt, Cardol, & Remacle, 2012), thereby allowing respiratory growth due to which complex I mutants display a characteristic slow‐growth‐in‐the‐dark (SID) phenotype. In a previous study by our group, a forward genetic screen conducted based on the SID phenotype led to the isolation of seven nuclear mutants, *amc1* to *amc7* (for *a*ssembly of *m*itochondrial *c*omplex I) defining six distinct loci required for complex I function (Barbieri et al., 2011). In this study, we report the description of *amc8* to *amc13* which were also uncovered via insertional mutagenesis. Among these mutants, the *amc5* and *amc9* mutations were mapped to nuclear genes encoding the complex I subunits NUOB10 (NDUFB10 in human) and NUO5 (NDUFV2 in human), respectively (Barbieri et al., 2011 and this study), proving the efficacy of our screen. We have utilized *Chlamydomonas* complex I mutants *amc5* (*nuob10/ndufb10‐null*) and *amc9* (*nuo5/ndufv2‐null*) as a platform for determining the pathogenicity of human mutations in the genes encoding NDUFB10 and NDUFV2, respectively. The human mutations were reconstructed in *Chlamydomonas*, and their effect on complex I activity and assembly were assessed.

## 2 MATERIALS AND METHODS

### 2.1 Strains and culture conditions

*Chlamydomonas* strains were grown in Tris‐acetate‐phosphate (TAP), with Hutner's trace elements, 20 mM Tris base and 17 mM acetic acid, or TAP supplemented with arginine (1.9 mM) (TARG), TARG supplemented with 25 µg/ml hygromycin B (TARG + HyB), or 25 µg/ml paromomycin (TARG + Pm) liquid or solid medium at 25°C in continuous light at 50 µmol m^−2^ s^−1^ (Harris, 1989). In accordance with our laboratory conditions, we define high light conditions as 50 µmol m^−2^ s^−1^ and low light conditions correspond to 0.5 µmol m^−2^ s^−1^. Solid medium contains 1.5% (w/v) select agar (Invitrogen, 30391049). The background strains used to generate transformants were 3A^+^ (*mt^+^*; *arg7‐8*) [CC‐5589] and 4C^−^ (*mt^−^*; *arg7‐8*) [CC‐5590] (Dr. Rochaix, University of Geneva). The strains 141 (*arg9‐2*; *mt^+^*), CC‐124 (*mt^−^*), CC‐125 (*mt^+^*), or 1’ (*mt^+^*) [a *137C* derivative, provided by Dr. Claire Remacle, University of Liège, Belgium] were used in crosses and/or as experimental controls. Strains *amc5* (87D3) [CC‐5591], *dum11* [CC‐4098], and *dum18* were used in this study (Barbieri et al., 2011; Remacle, Duby, Cardol, & Matagne, 2001a). Insertional mutagenesis and phenotypic screening of complex I mutants are detailed in Method S1. Genetic analyses are described in Method S2. Ten‐fold dilution series and growth curve analyses were conducted as described in Method S3.

*Saccharomyces cerevisiae* strain CW04 (*MATα ade2‐1 his3‐11,15 leu2‐3,11 trp1‐1 ura3‐1*; Banroques, Delahodde, & Jacq, 1986) was utilized for plasmid construction via gap repair (Method S5) and grown at 28°C in synthetic dextrose medium containing all amino acids (SD + AA) prior to plasmid construction. Colonies carrying the recombinant plasmids were selected in synthetic dextrose medium lacking only uracil (*SD*‐ura) (Dujardin, Pajot, Groudinsky, & Slonimski, 1980). Chemo‐competent *Escherichia coli* DH5α strains were used for molecular cloning. *E. coli* was grown at 37°C in Luria‐Bertani (LB) broth and agar (Silhavy, Berman, & Enquist, 1984).

### 2.2 TAIL‐PCR and PCR‐based screening of indexed cosmid library

Nucleic acid extraction, diagnostic PCRs, and real‐time quantitative PCRs were conducted as in Method S4.

TAIL‐PCR (thermal asymmetric inter‐laced PCR) was conducted to identify the sequence flanking the iHyg3 cassette (encoding the *APHVII *gene conferring hygromycin B resistance) in the *amc9* mutant as in Liu, Mitsukawa, Oosumi, and Whittier (1995) using the partially degenerate primer AD1 (Dent, Haglund, Chin, Kobayashi, & Niyogi, 2005; Liu et al., 1995; Table S1). The following iHyg3‐specific primers, APH7R3, APH7R4, and APH7R5 (Table S1), were used for the primary, secondary, and tertiary TAIL‐PCRs, respectively. Similar reactions were conducted using wild‐type genomic DNA and purified iHyg3 cassette to identify non‐specific amplification of DNA.

Cosmids containing *NUO5* and *NUOB10* genomic DNA were identified by PCR (Purton & Rochaix, 1994). The *NUO5‐*containing cosmid (referred to as 9A2) was identified using the primer pairs NUO5 E2L/NUO5 E3R (Table S1). The *NUOB10‐*containing cosmid (referred to as cosmid 7D10) was identified using the primer pairs NUOB10E1L/ NUOB10E4R (Table S1). The borders of *Chlamydomonas* genomic DNA inserted into these cosmids were sequenced to confirm the presence of genomic region including the gene of interest.

### 2.3 Biolistic transformation

The list of plasmids and recipient strains used for biolistic transformation is provided in Method S5 and Tables S2, S3, and S4. The recipient strains *amc9 (41D9) (mt^−^*; *nuo5::APHVII*; *arg7‐8)* [CC‐5601] or *amc5 (87D3) (mt^+^*; *nuob10::APHVIII*; *arg7‐8)* [CC‐5591] were subjected to biolistic transformation using a homemade particle delivery device. The recipient strain was grown in liquid TARG medium for 2–3 days until it reached the exponential phase (3–6 × 10^6^ cells/ml). The cells were plated on respective selective medium at 10^8^ cells/plate. For each bombardment, DNA was coated on sterile 0.6–0.9 µm tungsten particles (Strem Chemicals, # 93–7437) by using 2 µg of transforming DNA, 16.7 mM spermidine, and 1 M CaCl_2_. The bombardment was conducted at a helium pressure of 1.725 MPa and vacuum of ~92 kPa. The plate was positioned 10.5 cm away from the particle‐containing nozzle. The bombarded plates were first incubated at 0.5 µmol m^−2^ s^−1^ light overnight for recovery and then transferred to continuous light (50 µmol m^−2^ s^−1^). Transformants containing cosmids with the *ARG7* marker (9A2 for *amc9* and 7D10 for *amc5*) were selected based on arginine prototrophy. Transformants containing the mutant genes were selected based on their respective antibiotic resistance (Table S4) and were subsequently screened for the presence of the transgene by diagnostic PCR. The site‐directed mutations in each selected transformant were confirmed by sequencing.

### 2.4 Complex I activity measurements

Mitochondrial enzymatic activity measurements were conducted as described previously in Cardol, Matagne, and Remacle (2002), Remacle, Baurain, Cardol, and Matagne (2001b), Remacle, Gloire, Cardol, and Matagne (2004), with slight modifications. Cells grown for 2–3 days on solid medium were harvested and resuspended in MOPS‐KOH extraction buffer (10 mM MOPS‐KOH pH 7.4, 0.5 M mannitol, 100 mg/ml BSA, 0.5 mM PMSF). Cells were lysed by sonication using a Branson Sonifier 150 (1/8 inch probe tip), at 12 W output for 2 × 30 s. Following lysis, the extract was centrifuged at 480 *g* for 10 min, followed by 3,000 *g* for 5 min. The supernatant was centrifuged at 27,000 *g* for 20 min, and the resulting pellet was the crude membrane extract. Complex I activity was determined as the rate of NADH oxidation, which was measured spectrophotometrically at 340 nm. The substrates used were 100 µM NADH (Amresco, 0384‐1G) and 100 µM duroquinone (Aldrich, D22320‐4). Specific activity was calculated using the molar extinction coefficient for NADH at ε_340nm_ = 6.22 mM^−1^ cm^−1^ in the absence and presence of 45 µM rotenone (MP Biomedicals, 150154), a complex I‐specific inhibitor. Complex II + III and complex IV activity assays are described in Method S6.

### 2.5 Blue‐native PAGE (BN‐PAGE) and in‐gel activity assays

Partially purified membranes were extracted as described above for activity measurement. Complexes were separated by BN‐PAGE using 4%–12% (w/v) acrylamide gradient gels (Schägger & von Jagow, 1991). Membranes were partially solubilized as follows. Membrane proteins (500 µg) were pelleted at 18,000 *g* for 20 min at 4°C. The membranes were resuspended in 180 µl of 2% (w/v) sodium *n*‐dodecyl‐β‐D maltoside (DDM; Bioworld, 40430017‐3) and solubilized by incubating in DDM in wet ice for 1 hr, followed by addition of 20 µl of 10% (w/v) sodium taurodeoxycholate hydrate (TDC; Sigma, T‐0875). Both DDM and TDC were dissolved in ACA buffer (750 mM aminocaproic acid, 0.5 mM EDTA, 50 mM Bis‐Tris, pH 7). Partially solubilized membrane proteins (200 µg) were loaded per lane. *In‐gel* NADH dehydrogenase (complex I) activity was visualized as purple bands after incubating the gels in 100 mM MOPS‐KOH buffer, pH 8, containing 1 mg/ml *p*‐nitro blue tetrazolium chloride (NBT; GoldBio, NBT2.5) and 0.2 mM NADH. Following *in‐gel* complex I staining, *in‐gel* ATPase (complex V) activity was detected by incubating the gels overnight in the dark, in 50 mM HEPES‐KOH pH 8 buffer containing 30 mM CaCl_2_ and 8.2 mM ATP (Fisher Bioreagents, BP413‐25), until a white precipitate was visible. This precipitate revealed the ATPase activity of complex V. Coomassie staining was conducted for loading control. Immunoblotting methods are detailed in Method S7.

## 3 RESULTS

### 3.1 Isolation of novel complex I mutants via forward genetics

**Figure 1:**
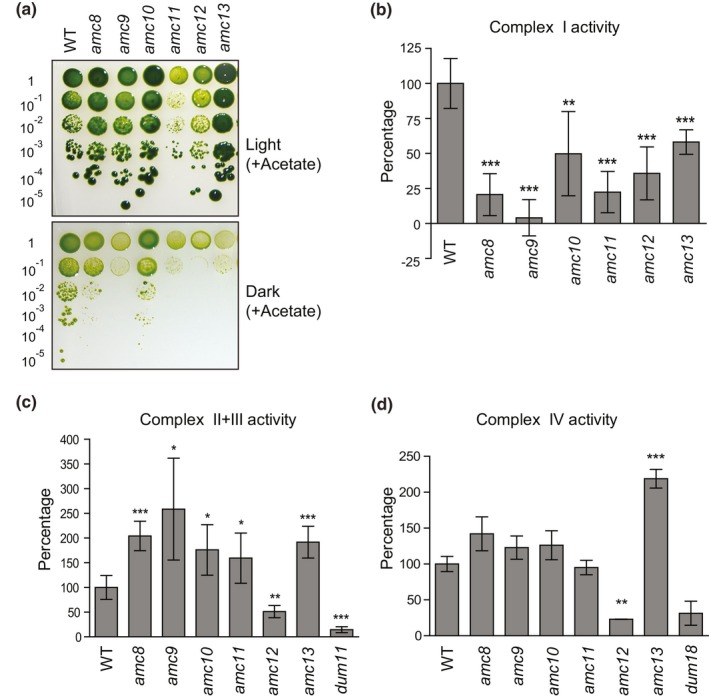
The *amc8* to *amc13* mutants exhibit complex I deficiency. (a) The growth phenotype of the wild‐type (WT, 4C^−^) and *amc8* to *amc13* mutants was analyzed by 10‐fold dilution series. The dilutions were plated on medium containing acetate as a carbon source and incubated in continuous light or in the dark for 20 days. In (b), (c), and (d), the enzymatic activities were conducted on crude membrane extracts and are displayed as percentage of the activity mean of WT, with the error bars indicating percentage of standard deviation of the mean. WT strain used for comparison is 4C^−^ for the *amc* strains and CC‐124 for *dum11* and *dum18*. Statistical significance was determined by two‐tailed unequal variances *t* test. * indicates *p* < .05, ** indicates *p* < .01, and *** indicates *p* < .001. (b) Complex I (rotenone‐sensitive NADH: duroquinone oxidoreductase) activity was determined from six independent biological replicates. The average complex I activity of WT was 46.6 ± 8.3 nmol NADH oxidized. min^−1^ mg^−1^ protein. (c) Complex II + III (succinate: cytochrome *c* oxidoreductase) activity was assessed from six independent biological replicates (except *amc8* for which five biological replicates were used). The WT displayed an activity of 18.1 ± 4.3 nmol of cytochrome *c* reduced. min^−1^ mg^−1^ protein. A mutant displaying complex III deficiency (*dum11*) was used as a control. (d) Complex IV activity (cytochrome *c* oxidase) was determined from three independent biological replicates. The WT displayed an activity of 269.6 ± 28.6 nmol of cytochrome *c* oxidized. min^−1^ mg^−1^ protein. A mutant displaying complex IV deficiency (*dum18*) was used as a control. In all the figures, the original mutant strains were used except for *amc10 (12C)* and *amc13 (16)* (in a, c, and d), which are derivatives of the original *amc10* and *amc13* mutants

To uncover additional *AMC* loci, insertional mutagenesis was conducted using the 4C^−^ wild‐type strain (*mt^−^*; *arg7‐8*) as the recipient and the iHyg3 cassette, encoding the *APHVII* gene that confers hygromycin B resistance (HyB^R^), as transforming DNA. The resulting transformants were screened by replica plating for the SID phenotype, a characteristic phenotype of complex I deficiency in *Chlamydomonas* (Remacle, Baurain, et al., 2001b). Among 4,200 insertional mutants, six *amc* mutants (*amc8* to *amc13*) displaying a SID phenotype (Figure 1a) and deficient in rotenone‐sensitive NADH: duroquinone oxidoreductase activity (Figure 1b), were isolated. While the *amc8*,* amc9*, and* amc11* strains had severely decreased complex I activity, the *amc10*,* amc12*, and *amc13* exhibited partial complex I deficiency. To test whether the mutation in the *amc* strains yielded defects in other respiratory enzymes, we measured complex II + III and IV activities (Figure 1c). Apart from *amc12*, all the *amc* mutants exhibited elevated complex II + III activity, a common feature previously observed in several *Chlamydomonas* complex I mutants (Barbieri et al., 2011; Cardol et al., 2002; Remacle, Baurain, et al., 2001b). None of the *amc* mutants, except *amc12*, displayed a defect in complex IV activity (Figure 1d). We concluded that all *amc* mutants, with the exception of *amc12*, displayed isolated complex I deficiency. The *amc12* mutant was pleiotropic with defects in complexes I, II + III, and IV.

### 3.2 The complex I mutants display defects in complex I assembly

**Figure 2:**
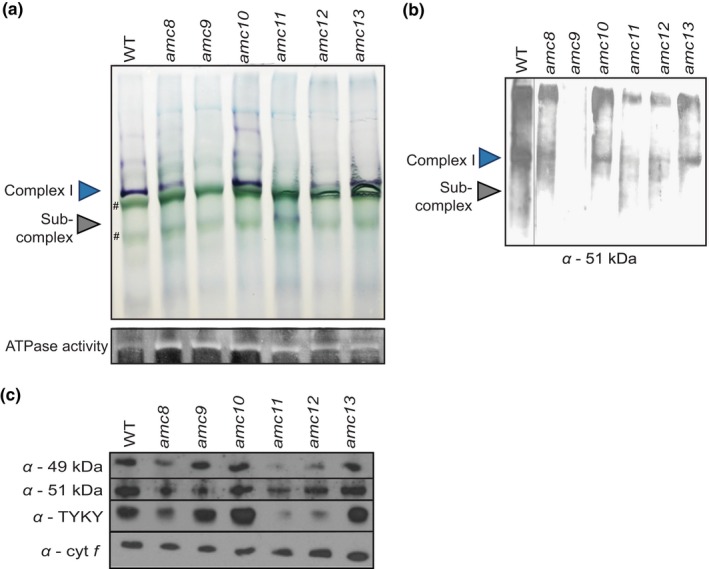
The *amc* mutants display a complex I assembly defect. (a and b) BN‐PAGE (blue‐native polyacrylamide gel electrophoresis) was conducted on 200 µg of partially purified membrane fraction. (a, *Top panel) In‐gel* complex I activity was detected by NBT staining. The purple bands indicate *in‐gel* staining of NADH dehydrogenase activity in mature (950 kDa) and partially assembled subcomplexes of complex I. In some cases, multiple purple bands larger than 950 kDa are detected, which could be due to partially solubilized membranes or might correspond to complex I in association with other complexes (Cardol et al., 2008). The symbol (#) indicates the photosynthetic complexes present in the crude membrane extract, migrating at sizes similar to that of the complex I holoenzyme and the subcomplex. They are marked only in two lanes for ease of reference. (a, *Bottom panel) In‐gel* ATPase staining to detect complex V was conducted to verify quality of crude membrane preparation and loading. The reduced ATPase staining in WT compared to the *amc* mutants was not systematically observed. (b) Immunoblotting was conducted, using α‐51 kDa antibody, on complexes separated by BN‐PAGE to detect the assembled soluble arm of complex I. This image is a composite of two gels run on the same day as indicated with a black vertical line: with the WT lane from one gel and the lanes corresponding to the *amc* mutants from another. (c) SDS‐PAGE immunoblotting was conducted on 10 µg of partially purified membranes using polyclonal antibodies to detect soluble arm complex I subunits: α‐49 kDa, α‐51 kDa, α‐TYKY. α‐cyt *f* was used to confirm equal loading. In (a) and (b), WT is the 4C^−^ strain and the *amc8* to *amc13* strains are the original mutants except the *amc10 (12C)* strain, which is a meiotic progeny derived from the original *amc10* strain

To assess the level of complex I assembly in the newly isolated *amc* mutants, protein complexes from crude membrane extracts were separated via BN‐PAGE (blue‐native polyacrylamide gel electrophoresis). Mature complex I (~950 kDa) and partially assembled subcomplexes were visualized by *in‐gel* staining that reveals NADH dehydrogenase activity as a purple band (Figure 2a). Note that complex I mutants with an assembled soluble arm are capable of *in‐gel* NADH oxidation, even if the ubiquinone reductase activity is impaired. Based on the *in‐gel* activity, we categorized the *amc* strains into four groups: (a) no active complex I in the *amc9* mutant, (b) accumulation of a subcomplex displaying NADH dehydrogenase activity in the *amc11* strain, (c) decreased levels of active complex in *amc8 and amc12*, and (d) wild‐type levels of *in‐gel* NADH dehydrogenase activity in *amc10* and *amc13* strains*.* BN‐PAGE immunoblotting analysis (Figures 2b and S1) showed that no assembled complex I was detected in *amc9*, whereas fully assembled complexes accumulating to a lesser degree than wild‐type were observed for *amc8*,* amc10*, *amc12*, and *amc13*. The highly labile subcomplex observed in *amc11* is indicative of a defect in assembling the distal membrane arm of complex I (Barbieri et al., 2011; Cardol et al., 2008).

It has been previously observed that some complex I mutants accumulate reduced levels of complex I subunits as a result of impaired holoenzyme assembly (Barbieri et al., 2011; Saada et al., 2008). Hence, the steady‐state accumulation of a subset of complex I subunits was examined by SDS‐PAGE immunoblotting analysis (Figure 2c). Three subunits from the soluble arm of complex I, 49 kDa (NUO7), 51 kDa (NUO6), and TYKY (NUO8) (Barbieri et al., 2011; Cardol, 2011), were chosen for analysis based on the availability of antibodies. Only *amc8*, *amc11*, and *amc12* accumulated reduced levels of 49 kDa, 51 kDa, and TYKY subunits, whereas *amc10* and *amc13* accumulated these subunits to wild‐type levels. The *amc9* mutant displayed decreased levels of the 51 kDa subunit.

### 3.3 The *amc9*, *amc11*, and *amc12* mutations are linked to the insertional marker

Although the complex I mutants were generated by insertional mutagenesis, previous genetic analyses of *amc* mutants showed that the insertional cassette is not always linked to complex I deficiency (Barbieri et al., 2011). The unlinked mutations could be due to the insertion of extracellular genomic DNA uptaken during electroporation (Zhang et al., 2014), insertion of cleaved and non‐functional pieces of the cassette, or insertion of the random herring sperm DNA used as part of the electroporation protocol (Shimogawara, Fujiwara, Grossman, & Usuda, 1998). Hence, genetic analyses were conducted to determine the nature of the *amc* mutations. Analysis of the heterozygous diploid progeny (*amc*/+) showed the diploids were restored for growth in the dark, indicating that all *amc* mutations were recessive for the complex I‐deficient phenotype (Figure S2). Meiotic progeny of *amc* × wild‐type crosses were tested to determine whether the *amc* mutations were monogenic and the insertional cassette co‐segregated with the complex I‐deficient phenotype (Table 1). The analyses indicated that the complex I deficiency in the *amc8*,* amc9*,* amc10*,* amc11*, and *amc13* strains exhibited monogenic inheritance (Table 1). In addition, all the HyB^R^ meiotic progeny, derived from genetic crosses of wild‐type, with *amc9*,* amc11*, or *amc12*, displayed a SID phenotype, indicating that the complex I deficiency in these mutants is tightly linked to the insertional cassette. On the other hand, for *amc8*,* amc10*,* and amc13*, only a fraction of the HyB^R^ recombinant meiotic progeny displayed the SID phenotype, indicating that the *AMC* locus responsible for the complex I‐deficient phenotype was segregating away from the antibiotic resistance insertional cassette. Tetrad analysis also confirmed that the complex I deficiency was not linked to the insertional cassette in the *amc10* and *amc13* mutants. We conclude that the recessive complex I deficiency in the *amc* mutants was linked to the insertional cassette only for *amc9*,* amc11*, and *amc12.* In this study, we show further characterization of the *amc9* mutant.

**Table 1:** Phenotypic and genetic analysis of the *amc* mutants

Strain	CI activity (%)	Fully assembled complex	Subcomplex	Genetic analysis	Recombinant meiotic progeny		Linkage to cassette	Monogenic
					Total	SID		
WT	100	++++	−	—	—	—	—	—
*amc8*	21	++	−	Bulk	230	50	No	Yes
*amc9*	4	−	−	Bulk, Tetrad	50	50	Yes	Yes
*amc10*	50	+++	−	Bulk, Tetrad	100	48	No	Yes
*amc11*	22	−	++	Bulk	100	100	Yes	Yes
*amc12*	36	++	−	Bulk	85	85	Yes	N.D.
*amc13*	58	+++	−	Bulk, Tetrad	112	51	No	Yes

Note

Complex I‐specific activity for the *amc* mutants was determined by measuring rotenone‐sensitive NADH: duroquinone oxidoreductase activity and is represented as percentage of WT activity (WT, 4C^−^ at 46.6 ± 8.3 nmol NADH oxidized. min^−1^ mg^−1^ protein, set to 100%). The detection of fully assembled complex and the subcomplex was determined from BN‐PAGE (blue‐native polyacrylamide gel electrophoresis) *in‐gel* activity and immunoblotting. ++++, +++, ++, +, − indicate relative levels of detected complex. To test whether the *amc* mutations are monogenic, genetic analysis of the meiotic progeny of *amc* x wild‐type crosses was performed by analyzing the 2:2 segregation of complex I phenotype in tetrads. In cases where tetrad analysis was not successful, bulk germination of zygotes was conducted and the resulting meiotic progeny were scored for complex I‐deficient phenotype. In the case of *amc9*, *amc10*, and *amc13*, each of the tetrads that were tested showed a 2:2 segregation of the SID (slow growth in the dark) and wild‐type heterotrophic growth phenotype, confirming monogenic inheritance of the complex I‐deficient trait (Figure S3). In the case of the *amc12* mutant, the monogenic inheritance of the SID phenotype could not be determined (N.D.) via tetrad analysis due to poor germination of the zygotes.

### 3.4 The *amc9* mutation maps to the *NUO5* gene encoding the 24 kDa subunit of complex I

In the case of *amc9*, analyses of seven tetrads and 50 HyB^R^ recombinant meiotic progeny obtained from bulk germination of meiotic zygotes showed that the antibiotic resistance always segregated with the SID phenotype (Figure S3). The tight linkage between the insertional marker and the SID phenotype in the *amc9* mutant suggests that the disruption of a gene controlling complex I, by the insertional cassette, could be responsible for the complex I deficiency. To identify the disrupted gene in the *amc9* mutant, we sought to recover the genomic sequence flanking the insertional cassette via TAIL‐PCR (Liu et al., 1995). The full‐length insertional cassette was mapped to exon 2 of the *NUO5* gene, which encodes the 24 kDa subunit (NUO5) of the soluble arm of complex I (Figure S4A,B) (Subrahmanian, Remacle, & Hamel, 2016)*.* Real‐time RT‐qPCR (Figure S4C) showed that the *amc9* mutant lacked the full‐length *NUO5* transcript. These results confirmed the insertion of a full‐sized iHyg3 cassette into exon 2 of the *NUO5* gene in the *amc9* mutant.

**Figure 3:**
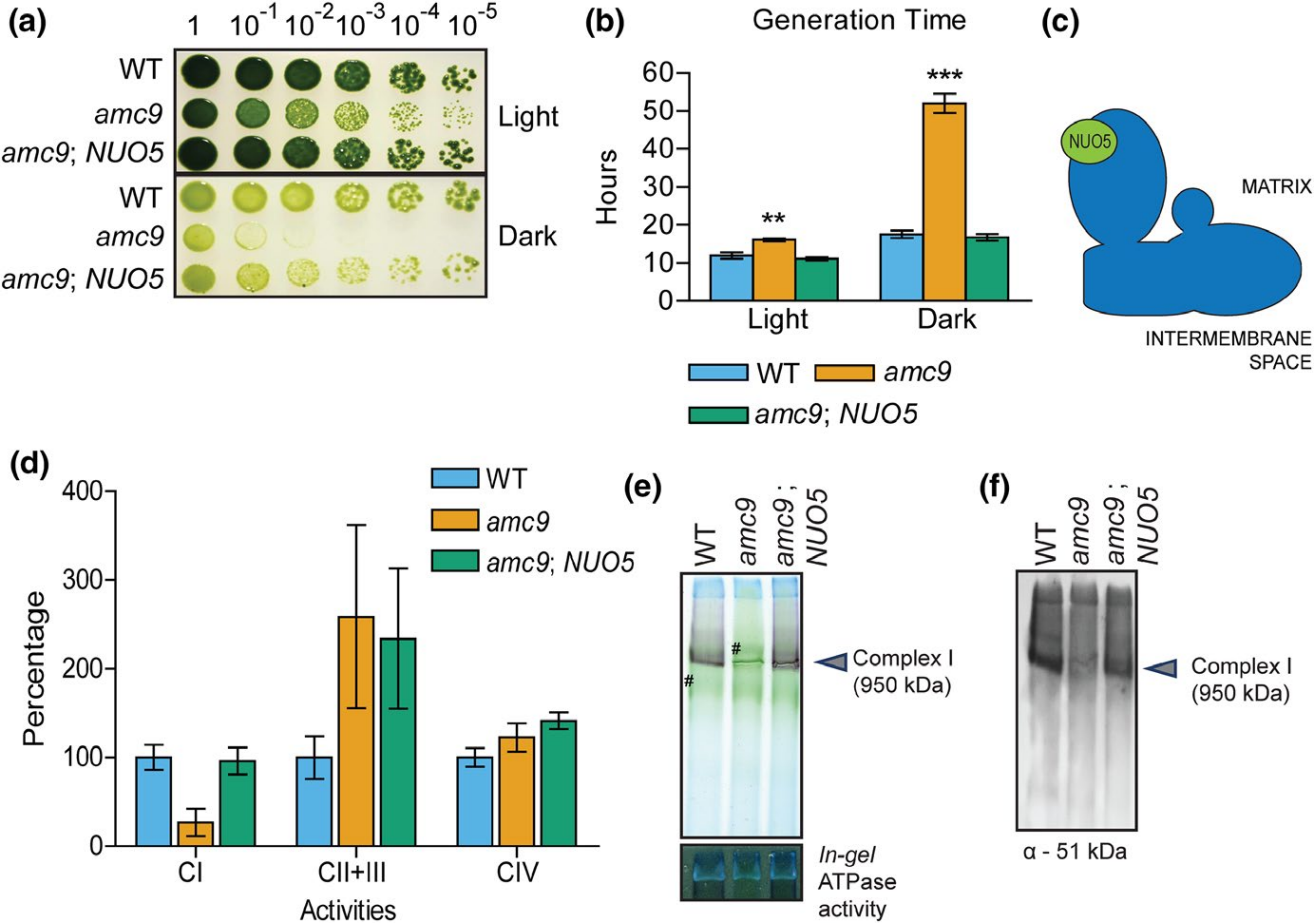
The *amc9* mutant, complemented by the *NUO5* gene, is restored for complex I activity and assembly. (a) Restoration of the growth phenotype in [*amc9*; *NUO5*] was tested by 10‐fold dilution series plated on acetate‐containing medium and incubated in the light for seven days and in the dark for 16 days. (b) The average generation time for each strain calculated from growth curves in Figure S4E is indicated here. The error bars represent standard deviation of the mean. Statistically significant difference with respect to the WT was determined by two‐tailed unequal variances *t* test. ** indicates *p* < .01, and *** indicates *p* < .001. (c) The approximate location of the NUO5 subunit in the matrix arm of complex I is indicated in a diagrammatic representation. (d) Complex I (CI), complex II + III (CII + III), and complex IV (CIV) activities were determined on partially purified membranes. The activities are represented as percentage of WT calculated from an average, with the error bars indicating standard deviation of the mean. The averages for CI, CII + III, and CIV activities were determined from three, six, and three biological replicates, respectively. For the reference strain, WT (4C^−^), average CI activity was 76.4 ± 19.9 nmol NADH oxidized. min^−1^ mg^−1^ protein, average CII + III activity was 18.1 ± 4.3 nmol cytochrome *c* reduced. min^−1^ mg^−1^ protein, and average CIV activity was 269.6 ± 28.6 nmol cytochrome *c* oxidized min^−1^ mg^−1^ protein. The *amc9* mutant displays a significant reduction in complex I activity with respect to WT, as determined by two‐tailed unequal variances *t* test with a *p* value = .03. The [*amc9*; *NUO5*] strain is rescued for complex I activity. While there is no significant difference between activities measured for the WT and [*amc9*; *NUO5*] strains, there is a significant difference between *amc9* and [*amc9*; *NUO5*] with a *p* = .019. (e) BN‐PAGE (blue‐native polyacrylamide gel electrophoresis) was conducted on 200 µg of partially purified membrane fraction. *In‐gel* complex I activity was detected by NBT staining. The symbol (#) indicates the chlorophyll‐containing complexes present in the crude membrane extract. *In‐gel* ATPase activity was detected with CaCl_2_/ATP staining. (f) BN‐PAGE followed by immunoblotting was conducted on 200 µg of partially purified membrane fraction using polyclonal antibody to detect the 51 kDa subunit of the soluble arm of complex I

To test whether the insertional mutation in the *NUO5* gene is responsible for the complex I defect, we transformed the *amc9* mutant with a cosmid containing the wild‐type copy of *NUO5* (referred to as [*amc9*; *NUO5*]) and assessed the recovery of complex I function. The *NUO5* transcript levels were restored upon complementation with the wild‐type *NUO5* gene (Figure S4C). Rescue of the growth phenotype in the [*amc9*; *NUO5*] strain was measured by assessing growth on solid medium and in liquid culture (Figures 3a,b and S4E). The generation time in the dark for wild‐type and *amc9* was 17.5 hr and 52 hr, respectively, whereas the [*amc9*; *NUO5*] strain displayed a wild‐type level of growth in the dark as evidenced by a generation time of 16.8 hr (Figure 3b). Further biochemical analyses of [*amc9*; *NUO5*] revealed wild‐type levels of NADH: duroquinone oxidoreductase activity (Figure 3d), complex I subunits abundance (Figure S4D), and complex I assembly (Figure 3e,f). From these results, we conclude that the *AMC9* locus corresponds to the *NUO5* gene encoding the 24 kDa complex I subunit (referred to as NUO5 in *Chlamydomonas* and NDUFV2 in humans [Pagniez‐Mammeri et al., 2012, Subrahmanian et al., 2016]).

### 3.5 The NDUFV2 K209R variant does not affect complex I activity or assembly in *Chlamydomonas*

NDUFV2/NUO5 is the 24 kDa soluble subunit localized to the matrix arm of the holoenzyme (Figure 3c) and is one of the core subunits harboring a 2Fe‐2S (N1a) cluster which is coordinated by four cysteines (Figure S5; Birrell, Morina, Bridges, Friedrich, & Hirst, 2013). As a highly conserved protein, human NDUFV2 displays 51% identity with the *Chlamydomonas* NUO5 ortholog (Figure S5) and is a known marker for complex I disorders (Pagniez‐Mammeri et al., 2012). While the association of complex I deficiency with Parkinson's disease (PD) is a well‐established phenomenon, the exact molecular mechanisms defining how specific complex I‐related mutations cause pathogenesis have remained unclear (Giachin et al., 2016). One particular lysine‐to‐arginine (K209R) variant in NDUFV2 was detected in one out of 33 familial probands and one out of 238 sporadic PD cases (Nishioka et al., 2010). However, complex I enzymatic activity was not assessed in these patients to determine whether this mutation causes a complex I deficiency that may contribute to the development of PD.

In some cases, it has been shown that lysine‐to‐arginine substitutions may affect protein folding, and in others, it has been proposed that lysine‐to‐arginine substitutions increase stability through putative salt bridges and hydrogen bond formations (Sokalingam, Raghunathan, Soundrarajan, & Lee, 2012). To investigate the biochemical effect of the NDUFV2‐K209R variant on complex I activity and assembly, the corresponding mutation was reconstructed in the gene encoding the *Chlamydomonas* NUO5 ortholog and the variant was expressed in the *nuo5‐null* mutant strain *amc9*. This lysine residue is well‐conserved in eukaryotic species and occurs at position 230 of *Chlamydomonas* NUO5 (yellow highlight, Figure S5). The sequence encoding either the wild‐type or the K230R subunit was introduced into a construct containing the *NUO5* genomic DNA, fused to a sequence encoding a C‐terminal FLAG‐tag, under the control of the *NUO5* native promoter. Transformants were generated in the *amc9* strain via biolistics, and those accumulating the FLAG‐tagged NUO5 protein were selected for further analyses (Figure S6).

**Figure 4:**
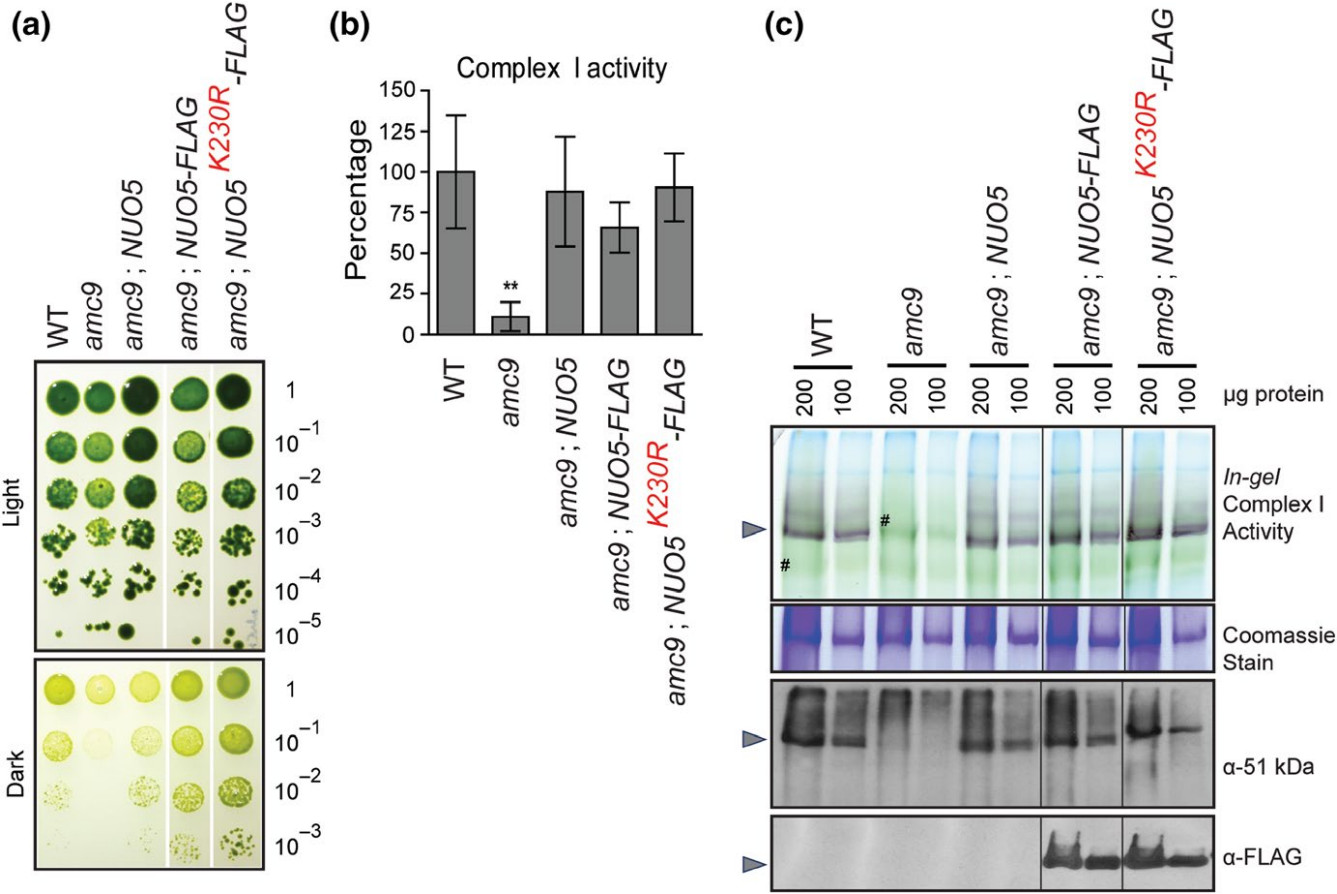
The lysine‐to‐arginine substitution in NUO5 does not affect complex I activity and assembly. (a) The growth phenotype of the WT and the *amc9* transformants was analyzed by 10‐fold dilution series plated on acetate‐containing medium and incubated in the light or in the dark for 14 days. White vertical lines indicate strains tested on the same plate and assembled together for this figure. (b) Complex I activity was determined with partially purified membranes from four biological replicates and represented as a percentage of WT average with the error bars indicating standard deviation of the mean. For WT (4C^−^), average complex I activity was 71 ± 24.7 nmol NADH oxidized. min^−1^ mg^−1^ protein. The *amc9* mutant displayed a significant reduction in complex I activity with respect to WT, as determined by two‐tailed unequal variances *t* test with a *p* value = 0.005. The [*amc9*; *NUO5‐FLAG*] and the [*amc9*; *NUO5^K230R^‐FLAG*] transformants were restored for complex I activity. (c) BN‐PAGE (blue‐native polyacrylamide gel electrophoresis) was conducted on 200 µg and 100 µg of partially purified membrane fraction. *In‐gel* complex I activity was detected by NBT staining. The gray arrowheads indicate fully assembled holoenzyme. The green bands (indicated with the symbol #) correlate to the co‐purified photosynthetic complexes in the membrane fractions. Coomassie staining following BN‐PAGE was used to test for equal loading. Protein(s) migrating at a size unrelated to complex I is shown here for this purpose. BN‐PAGE followed by immunoblotting was conducted using a polyclonal antibody to detect the 51 kDa subunit of the soluble arm of complex I and a monoclonal α‐FLAG antibody to detect NUO5‐FLAG. Black vertical thin lines indicate lanes assembled together from the same gel/blot. The NUO5^K230R^‐FLAG is incorporated into the fully assembled complex I and allows wild‐type levels of NADH dehydrogenase activity as detected by *in‐gel* complex I activity assay

The potential impact of the lysine‐to‐arginine substitution on growth was assessed (Figure 4a). As observed previously, the *amc9* mutant strain exhibited a SID phenotype whereas complementation with the wild‐type *NUO5* gene or recombinant *NUO5* gene with a C‐terminal FLAG‐tag restored growth in the dark to wild‐type levels. Interestingly, transformants expressing the K230R NUO5‐FLAG variant also exhibited wild‐type growth in the dark, indicating that this substitution in NUO5 does not affect respiratory growth (Figure 4a). Accordingly, NADH: duroquinone oxidoreductase activity was also restored by the K230R NUO5 variant (Figure 4b). To test the level of complex I assembly in the NUO5 lysine‐to‐arginine variant, complexes were separated by BN‐PAGE and subjected to *in‐gel* activity assays and immunoblotting (Figure 4c). The NUO5 subunit is part of the matrix arm responsible for NADH dehydrogenase activity. As a result, no mature complex I or subcomplexes were detected by *in‐gel* activity or immunoblotting in the *amc9* mutant. Complementation with the wild‐type *NUO5‐FLAG* gene restored complex I assembly. In addition, immunoblotting with the α‐51 kDa or α‐FLAG antibodies showed that the NUO5‐FLAG K230R variant was successfully incorporated into the holoenzyme. This was in agreement with detection of fully assembled complex I via *in‐gel* activity assay showing that the NADH dehydrogenase activity of the soluble arm was restored. From these results, we conclude that the K230R substitution in NUO5, a candidate mutation for PD, does not affect complex I activity or assembly in *Chlamydomonas.*

### 3.6 The *AMC5* locus corresponds to the NUOB10‐encoding gene

**Figure 5:**
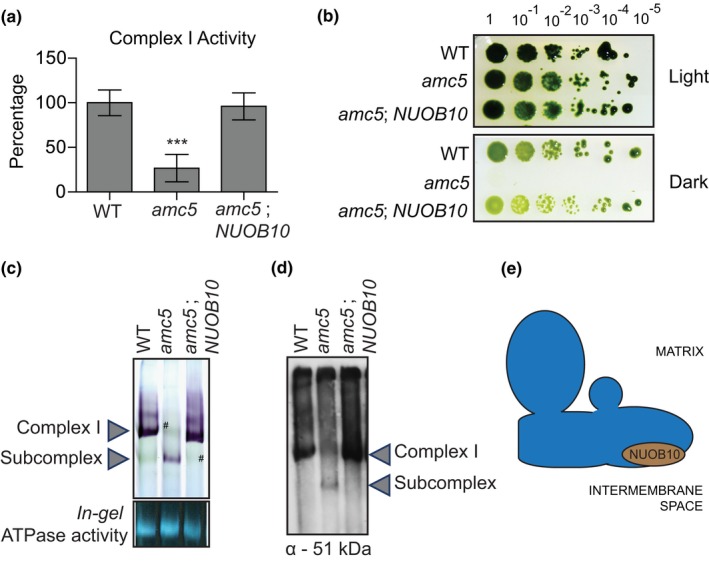
The *amc5* mutant phenotypes are rescued by the *NUOB10* gene. The *amc5* strain was transformed with a *NUOB10‐*containing cosmid by biolistics. The phenotypic rescue of one representative transformant [*amc5*; *NUOB10*] is shown here. The WT and *amc5* strains shown here are 3A^+^ and *amc5 (87D3)*, respectively. (a) Complex I (rotenone‐sensitive NADH: duroquinone oxidoreductase) activity was determined with partially purified membranes. The activities are represented as a percentage of the average of three biological replicates with the error bars indicating percentage of standard deviation of the mean. The average complex I activity of WT is 54.0 ± 7.7 nmol NADH oxidized. min^−1^ mg^−1^ protein. The activities for the WT and *amc5* strains are significantly different according to the two‐tailed unequal variances *t* test with a *p* = .000116. The *amc5* and [*amc5*; *NUOB10*] strain have significantly different activities with a *p = *.000199. (b) Restoration of the growth phenotype in [*amc5*; *NUOB10*] was tested by 10‐fold dilution series plated on acetate‐containing medium and incubated in the light for seven days and in the dark for 16 days. (c and d) BN‐PAGE was conducted on 200 µg of partially purified membranes. (c) *In‐gel* complex I activity was detected by NBT staining, and *in‐gel* ATPase activity was detected with CaCl_2_/ ATP staining. Crude membrane extracts, also containing the photosynthetic membranes, were used for this analysis. The symbol (#) indicates examples of photosynthetic complexes migrating closely with complex I and subcomplex that can be clearly observed in their absence. (d) Immunoblotting was conducted using a polyclonal antibody to detect the 51 kDa subunit of complex I. (e) A diagrammatic representation of the L‐shaped mitochondrial complex I, with the approximate location of the NUOB10/NDUFB10 subunit in the distal membrane arm facing the IMS (Zhu et al., 2015b)

NUOB10/NDUFB10/PDSW is an accessory subunit that is localized to the distal end of the membrane arm, although NUOB10 is hydrophilic and does not contain any predicted transmembrane helices (Hirst, Carroll, Fearnley, Shannon, & Walker, 2003) (Figures 5e and S7). It is predicted to face the intermembrane space (IMS) and presumably associated with the membrane through interactions with neighboring membrane subunits (Zhu et al., 2015a). The *Chlamydomonas* NUOB10 displays 13.5% identity with the human ortholog NDUFB10.

The *amc5* mutant harbors the insertional cassette in intron 3 of the *NUOB10* gene (Barbieri et al., 2011) accompanied by a deletion of the *NUOB10* genomic sequence downstream of the insertion site (Figure S8A,B). The *amc5* mutant displayed decreased rotenone‐sensitive NADH: duroquinone oxidoreductase activity (Figure 5a) and exhibited the characteristic *sid* phenotype of complex I‐deficient mutants in both liquid and solid medium (Figures 5b and S8D,E) with an average generation time of 69 hr in the dark, compared to 27 hr for the wild‐type strain. Real‐time RT‐qPCR confirmed the loss of the wild‐type *NUOB10* mRNA in the *amc5* mutant (Figure S8C). The *amc5* mutant displayed an accumulation of a subcomplex, migrating at a size similar to the ~700 kDa subcomplex previously observed in *Chlamydomonas* mitochondrial mutants defective for the distal membrane arm assembly of complex I (Cardol et al., 2008; Remacle et al., 2008; Figure 5c,d).

To test whether the mutation in *NUOB10* is indeed responsible for the complex I defect, the *amc5* mutant was transformed with a cosmid containing the *NUOB10* gene (Figures 5 and S8). Molecular analyses of the [*amc5*; *NUOB10*] transformant revealed the presence of the wild‐type *NUOB10* gene and restoration of relative *NUOB10* transcript levels. The [*amc5*; *NUOB10*] strain also exhibited restoration of growth in the dark, complex I activity, and assembly. From these results, we conclude that the *AMC5* locus corresponds to the *NUOB10* gene and the NUOB10 subunit is necessary for complex I membrane arm assembly.

### 3.7 The NUOB10 C‐(X)_11_‐C motif is important for complex I activity and assembly

To date, only one patient has been reported with mutations in *NDUFB10.* The patient, born to non‐symptomatic parents, exhibited fetal cardiomyopathy and fatal infantile lactic acidosis, and died at 27 hr after birth (Friederich et al., 2017). Exome sequencing identified compound heterozygous sequence variation in the *NDUFB10* gene: (a) a paternally inherited nonsense mutation resulting in a premature stop codon, and (b) a maternally inherited missense mutation resulting in a cysteine‐to‐serine (C107S) substitution. This cysteine is part of a highly conserved C‐(X)_11_‐C motif (yellow highlight, Figure S7), whose function in complex I activity and assembly has not been elucidated.

To understand the effect of the C107S substitution in NDUFB10 on complex I holoenzyme, we sought to reconstruct the corresponding mutation in the *Chlamydomonas nuob10‐*null *amc5* mutant*.* The C107 residue in human NDUFB10 corresponds to the first cysteine of the C‐(X)_11_‐C motif, at position 79 in the *Chlamydomonas* NUOB10 ortholog. To further gain insight into the role of the C‐(X)_11_‐C motif in complex I activity, a cysteine‐to‐serine substitution at the second cysteine of the motif (C91S) and the double substitution (C79S‐C91S) were also tested. For this purpose, the *NUOB10* genomic sequence (corresponding to wild‐type, C79S, C91S, C79S‐C91S variants) was fused to a sequence encoding a C‐terminal FLAG‐tag and expressed under the control of its native promoter. The *NUOB10* constructs were introduced into the *amc5/nuob10‐*null mutant strain by biolistics. Transformants carrying the transgene were identified and chosen for further analyses (Figure S9A). To test the accumulation of NUOB10‐FLAG in the transformants, immunoblotting was conducted. The NUOB10‐FLAG variants were detected at the expected size of ~17 kDa with the α‐FLAG antibody (Figure S9B).

**Figure 6:**
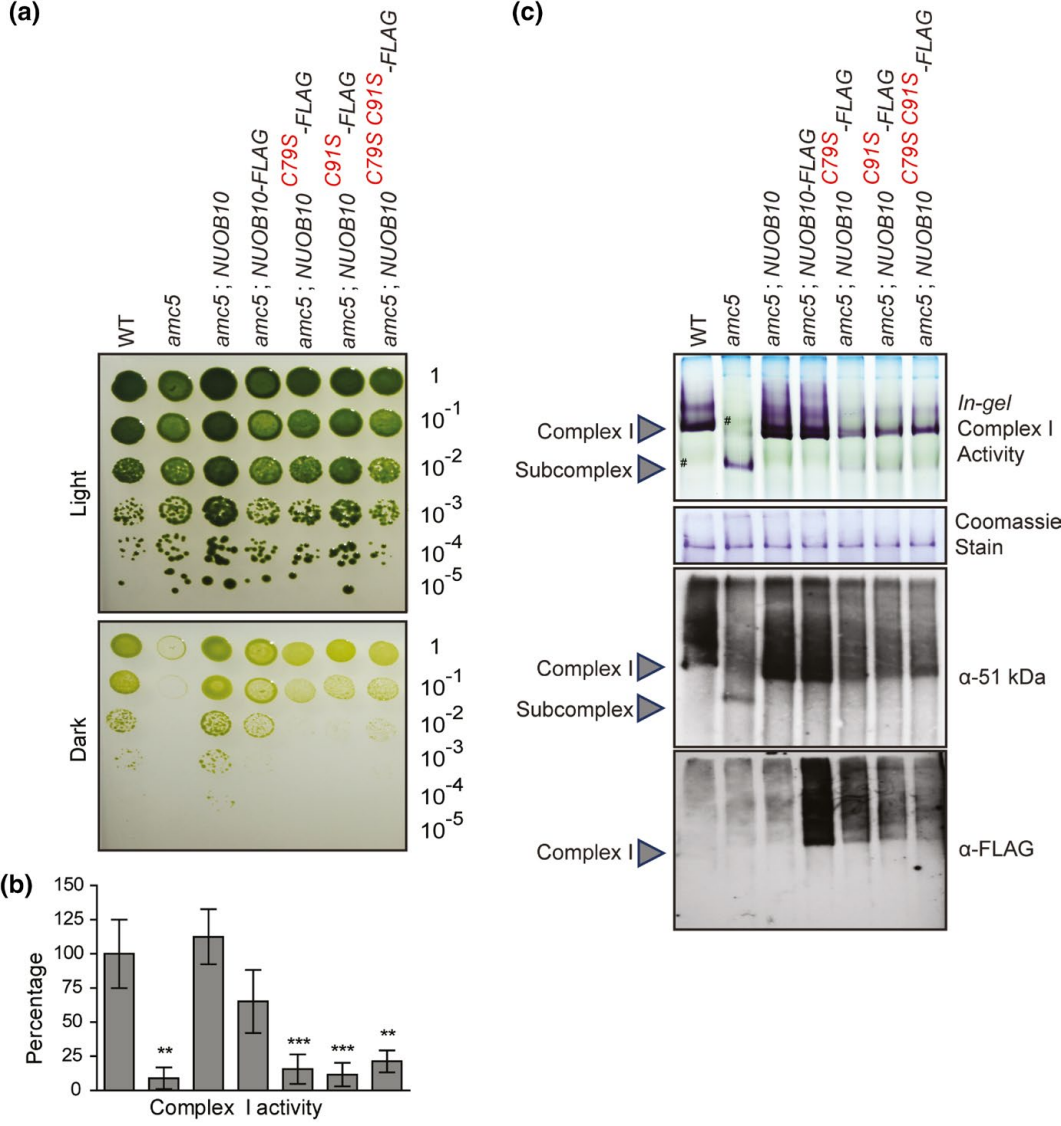
The cysteine‐to‐serine substitutions in NUOB10 decrease complex I activity and assembly. The *amc5* mutant was transformed with four constructs containing the *NUOB10* genomic DNA: (i) wild‐type *NUOB10* sequence (*NUOB10* or *NUOB10‐FLAG*), and mutant *NUOB10* sequences encoding the variants with (ii) the C79S substitution (*NUOB10^C79S^‐FLAG*), (iii) the C91S substitution (*NUOB10^C91S^‐FLAG*), or (iv) the C79S and C91S double substitutions (*NUOB10^C79SC91S^‐FLAG*). The wild‐type (WT, 3A^+^), *amc5* mutant, and [*amc5*; *NUOB10*] strains were used as controls. (a) The growth phenotype of the wild‐type and *amc5* transformants was analyzed by 10‐fold dilution series. The dilutions were plated on medium containing acetate as a carbon source and incubated in the light or in the dark for 14 days. (b) Complex I activities, conducted with partially purified membranes, are represented as percentage of WT calculated from the average of five biological replicates with the error bars indicating standard deviation of the mean. For WT, average complex I activity was 48.3 ± 12.1 nmol NADH oxidized. min^−1^ mg^−1^ protein. The *amc5* transformants producing the C79S, C91S, and C79SC91S variants display significantly reduced complex I activities compared to WT as determined by two‐tailed unequal variances *t* test. ** indicates *p* < .01, and *** indicates *p* < .001. The graph is aligned to match with the labels in (a). (c) BN‐PAGE was conducted on 200 µg of partially purified membrane proteins. *In‐gel* complex I activity was detected by NBT staining. The symbol (#) indicates the photosynthetic complexes present in the crude membrane extract. Although present in all the lanes, they are marked only in two lanes for ease of reference. Coomassie staining serves as loading control, and protein(s) migrating at a size different from complex I is shown here. Immunoblotting was conducted using a polyclonal antibody to detect the 51 kDa subunit of the soluble arm of complex I and a monoclonal α‐FLAG antibody to detect the NUOB10‐FLAG

The effect of the NUOB10 cysteine substitutions on respiratory growth was tested (Figure 6a). While the *amc5* recipient strain displayed a SID phenotype, transformants expressing the wild‐type *NUOB10‐FLAG* had restored growth in the dark similar to those with the *NUOB10‐*containing cosmid. On the other hand, the single and double cysteine‐to‐serine variants displayed only partial restoration of growth in the dark. These observations indicate that while expression of the *NUOB10* variants can partially compensate for loss of NUOB10, manipulation of the C‐(X)_11_‐C motif restricts respiratory growth.

To test the importance of the C‐(X)_11_‐C motif for complex I activity and assembly, rotenone‐sensitive NADH: duroquinone oxidoreductase activity and BN‐PAGE *in‐gel* activity were assessed (Figure 6b,c). The *amc5* strain transformed with wild‐type *NUOB10‐FLAG* showed rescue of complex I activity to ~65% of wild‐type levels (Figure 6b). Although the restoration of complex I activity for the [*amc5*; *NUOB10‐FLAG*] transformant was not as high as with the [*amc5*; *NUOB10*] control strain, BN‐PAGE *in‐gel* activity and immunoblotting revealed wild‐type levels of mature complex I at ~950 kDa, showing that the C‐terminal FLAG‐tag does not significantly impair complex I assembly (Figure 6c). On the other hand, the cysteine‐to‐serine single and double substitutions in NUOB10 yielded severe complex I deficiency, indicating that the C‐(X)_11_‐C motif is crucial for complex I activity (Figure 6b). Interestingly, the accumulation of the 700 kDa subcomplex due to the loss of NUOB10 in the *amc5* mutant was attenuated in the presence of the NUOB10 variants (Figure 6c). Mature complex I (~950 kDa), absent in the *amc5* mutant, was detected in the single and double cysteine‐to‐serine variants via *in‐gel* activity and immunoblotting with α‐51 kDa. Immunoblotting with α‐FLAG (detecting NUOB10) further revealed that the NUOB10 variants were incorporated into the mature complex. The restoration of complex I assembly, in spite of the cysteine‐to‐serine substitutions, could account for the slight improvement in the complex I activity levels of the cysteine‐to‐serine variants (~16% of wild‐type) compared to the *amc5* mutant (~8% of wild‐type) and partial rescue of the respiratory growth phenotype (Figure 6a,b). However, the NUOB10 variants failed to accumulate wild‐type levels of mature complex I, as evidenced by immunoblotting (Figure 6b). The level of complex I assembly is similar for the single or double mutant variants, an indication that mutation of either or both cysteines of the C‐(X)_11_‐C motif elicits the same impact on complex I activity and assembly. Additionally, the three distinct NUOB10‐FLAG cysteine‐to‐serine variants accumulated to only 50% of wild‐type (Figure S9B), indicating that the cysteines may also be required for the stability of NUOB10. From these observations, we concluded that the cysteines within the highly conserved C‐(X)_11_‐C motif of NUOB10 play an important role in complex I assembly and activity.

## 4 DISCUSSION

Mitochondrial complex I, the first and largest enzyme of the mitochondrial ETC, is a proton‐pumping NADH: ubiquinone oxidoreductase (Remacle et al., 2012). In an effort to isolate novel mutants for unraveling complex I biogenesis, a forward genetic approach in *Chlamydomonas reinhardtii* was undertaken. In the first part of this study, we described the isolation of six complex I mutants *amc8* to *amc13*, in addition to the previously characterized *amc1* to *amc7* (Barbieri et al., 2011). We showed that, except for *amc12*, the *amc* mutants displayed isolated complex I deficiency (Figure 1) with various levels of assembly defects of the mutants (Table 1; Figure 2). The *amc9* mutation resulted in no detectable complex, whereas the *amc11* mutation caused the accumulation of a subcomplex*—*both indicative of the assembly process being prematurely aborted. On the other hand, *amc8*,* amc10*,* amc12*, and *amc13* mutants were capable of assembling a mature holoenzyme.

So far, the forward genetic screen conducted by our group has resulted in the isolation of 12 complex I mutants from ~54,000 insertional mutants (Barbieri et al., 2011 and this study). Two *AMC* loci (*AMC9* and *AMC5)* encode for complex I subunits, verifying that the forward genetic screen yields *bona fide* complex I mutants. The other *amc* mutations remain yet‐to‐be determined and could map to any of the numerous genes encoding either complex I subunits or biogenesis factors. Considering the number of proteins required for complex I biogenesis (Subrahmanian et al., 2016), it is clear our forward genetic screen is far from saturated. Screening for the *sid* phenotype appears to have only a 0.02% frequency of obtaining a *bona fide* complex I mutant. The use of a larger insertional mutant library, similar to the CLiP library generated by the Jonikas group (Zhang et al., 2014), could yield additional novel *AMC* loci. Unfortunately, in our experience, the conditions used for generating the CLiP mutants are more conducive for isolating photosynthetic‐deficient mutants and less so for respiratory‐deficient mutants. The Remacle group has devised a new method of screening for respiratory mutants that is based on the concerted contribution of the photosynthetic and respiratory systems to cellular ATP production (Massoz et al., 2017, 2015). They used the *pgrl1* mutant, defective for photosystem I cyclic electron transfer that is involved in ATP production in chloroplasts, as the background for generating respiratory mutants. Respiratory deficiency in the *pgrl1* background displayed an additional phenotype defined by decreased photosystem II efficiency, which was used as the basis to screen for complex I‐deficient nuclear mutants (Massoz et al., 2017, 2015). Again, only three out of 3,059 transformants (0.09%) were identified as true complex I mutants from this screen. Future development of a screen to positively select or enrich for complex I mutants after mutagenesis might increase the success rate, yielding a larger number of nuclear mutants deficient for complex I.

*Chlamydomonas* has been previously used as a successful tool for studying human mitochondrial mutations. The L158P substitution in the mitochondrially encoded ND4 subunit, observed in one patient with chronic progressive external ophthalmoplegia (Pulkes, Liolitsa, Nelson, & Hanna, 2003), was shown to affect complex I activity but not assembly when reconstructed in *Chlamydomonas* (Larosa, Coosemans, Motte, Bonnefoy, & Remacle, 2012)*.* In the second part of this study, we exploited the high degree of conservation *of Chlamydomonas* complex I nuclear‐encoded subunits with their human counterparts (Cardol, 2011; Cardol et al., 2004) and demonstrated the efficacy of utilizing the newly uncovered *Chlamydomonas* nuclear mutants as a tool for defining the consequence of potentially pathogenic human mutations on complex I assembly and activity.

Both NUO5 (NDUFV2 in human) and NUOB10 (NDUFB10 in human) are highly conserved complex I subunits that have been identified as critical markers in human mitochondrial disorders (Benit et al., 2003; Cameron et al., 2015; Friederich et al., 2017; Nishioka et al., 2010; Zhang et al., 2009). NDUFV2 has been implicated in Alzheimer's disease, bipolar disorder, Parkinson's disease, and other pathologies. In this study, we tested a provisional mutation causing a lysine‐to‐arginine substitution in NDUFV2 and showed that it does not affect complex I activity or assembly. Indeed, comparative analyses of NDUFV2 orthologs revealed that an arginine residue is present at this position in *Thermus thermophilus*,* Arabidopsis*, and *Vitis vinifera* (Figure S5). As both lysine and arginine are positively charged amino acids, making the substitution of a conservative nature, it is likely this particular substitution is well‐tolerated and does not elicit a change in complex I activity in *Chlamydomonas*. While complex I is a highly conserved enzyme in all eukaryotes, it is expected that some differences do exist between organisms regarding the point of entry of subunits during the assembly process (Vogel, Smeitink, & Nijtmans, 2007). Hence, we cannot rule out that the lack of an effect on complex I due to the lysine‐to‐arginine substitution is *Chlamydomonas*‐specific. Three other SNPs in the *NDUFV2* gene resulting in amino acid substitutions of uncertain significance have been recently listed in the ClinVar database: P139T, M185V, and D190G. The P139T and M185V are especially interesting substitutions as these residues are very close to the cysteines involved in binding the Fe‐S cluster (Figure S5). A future line of investigation could be to test the importance of these residues with our *nuo5‐*null mutant.

The first patient reported with an isolated complex I disorder due to a mutation in the human nuclear *NDUFB10* gene presented an early‐onset phenotype, characterized by prenatal cardiomyopathy along with metabolic acidosis and failure to thrive (Friederich et al., 2017). In concert with a compound heterozygous nonsense mutation, a missense mutation characterized by a cysteine‐to‐serine substitution of the first cysteine in the C‐(X)_11_‐C motif resulted in decreased levels of complex I activity and increased accumulation of assembly intermediates in skeletal muscle, heart, and liver tissues (Friederich et al., 2017).

Recently, NDUFB10 was identified as an interacting partner of CHCHD4, a disulfide bond‐forming enzyme, via affinity purification in both denaturing and native conditions, implying that NDUFB10 could be an in vivo target of CHCHD4 (Petrungaro et al., 2015). The CHCHD4‐ALR import machinery (Mia40‐Erv1 in yeast), also known as the mitochondrial IMS assembly system (MIA), functions by interacting with the cysteine residues of the substrate proteins and driving their import from the outer mitochondrial membrane into the IMS by coupling translocation with disulfide bond formation (Gabriel et al., 2007; Herrmann & Riemer, 2014). Canonical CHCHD4 substrates contain twin C‐(X)_3_‐C or C‐(X)_9_‐C motifs, whose cysteines form intramolecular disulfide bonds spanning the two motifs, enabling the formation of an anti‐parallel helix–turn–helix structure (Herrmann & Riemer, 2014). The complex I subunits NDUFS5, NDUFB7, and NDUFA8 are canonical substrates of the CHCHD4‐MIA system containing twin C‐(X)_9_‐C motifs (Szklarczyk et al., 2011). Interestingly, the human NDUFB10 protein contains five cysteines in non‐canonical motifs: a C‐(X)_6_‐C motif, the C‐(X)_11_‐C motif, and a fifth single cysteine (Figure S7). Pulse‐chase experiments showed that cysteine thiols in NDUFB10 are no longer free after import into isolated human mitochondria and therefore presumed to be disulfide‐linked (Friederich et al., 2017). It is expected that two disulfide bonds are formed in NDUFB10 upon import (Friederich et al., 2017). Although the identity of the disulfide bond‐forming cysteines remains to be biochemically ascertained, single‐particle electron cryo‐microscopy of fungal, murine, bovine, and ovine complexes I model a disulfide bond between the cysteines within each motif of the NDUFB10 subunit (Agip et al., 2018; Letts, Fiedorczuk, Degliesposti, Skehel, & Sazanov, 2019; Parey et al., 2018; Zhu, Vinothkumar, & Hirst, 2016). As NDUFB10 does not have a canonical mitochondrial targeting sequence that is cleaved upon import (Hirst et al., 2003) and NDUFB10 sulfhydryl oxidation was shown to be CHCHD4‐dependent, it was hypothesized to be imported to the IMS via the oxidative folding MIA mechanism (Friederich et al., 2017). In comparison with NDUFB10, the *Chlamydomonas* NUOB10 and other vascular plant orthologs contain only the C‐(X)_11_‐C motif (Figure S7). If the cysteines are vital for mitochondrial import via the conserved oxidative folding mechanism, we should expect cysteine‐to‐serine substitutions of this motif to abolish NUOB10 import into the *Chlamydomonas* mitochondria and subsequent assembly into complex I. On the contrary, we observed that the single and double substitutions still allowed for incorporation of the NUOB10 variants into complex I, resulting in the accumulation of a mature holoenzyme (Figure 6). This finding is in accordance with the observations in the C107S NDUFB10 patient tissues, where complex I deficiency was not consistent across all tissues. For example, the skin fibroblasts appeared to express more of the C107S NDUFB10 variant than other tissues, enabling normal range of complex I activity (Friederich et al., 2017). These results indicate that the C107S substitution did not abolish NDUFB10’s import into human mitochondria. Instead, the degree of complex I deficiency in different tissues was due to tissue‐specific differential expression of this C107S variant. Our results further emphasize that both cysteine residues of the C‐(X)_11_‐C motif are not strictly essential for NUOB10 import into the *Chlamydomonas* mitochondria.

One alternative explanation for the above‐mentioned observations is that NUOB10/NDUFB10 is imported into the mitochondria in a CHCHD4‐dependent but cysteine‐independent manner. Such a phenomenon has been described for the IMS‐localized mitochondrial protease Atp23 (Weckbecker, Longen, Riemer, & Herrmann, 2012), wherein the disulfide bonds are required for protein folding and stability, instead of mitochondrial import (Weckbecker et al., 2012). Atp23 is imported via hydrophobic interactions with CHCHD4, even in the absence of all cysteine residues. Interestingly, the yeast Atp23 contains ten cysteines, including one possible C‐(X)_11_‐C motif similar to NDUFB10. All ten cysteines are involved in disulfide bond formation in the IMS, although the identity of the disulfide bond‐forming cysteine residues remains unknown. A second possibility is that NUOB10 could be imported into the IMS via an alternative import mechanism independent of the MIA machinery, and is instead involved in post‐import interaction with CHCHD4. For instance, it could be localized to the mitochondria by virtue of unknown internal targeting sequence(s) present in the protein, as is the case for BCS1, a factor required for complex III maturation (Stan et al., 2003). On the other hand, MICU1, another non‐canonical substrate of the MIA machinery, contains a mitochondrial targeting sequence and is imported through a CHCHD4‐independent transport. Post‐import, CHCHD4 interacts with its substrate MICU1, catalyzing intermolecular disulfide bond formation, which enables the assembly of MICU1 into the mitochondrial calcium uniporter complex (Petrungaro et al., 2015).

Because the cysteine variants of NUOB10 are still incorporated into complex I, the subunit does not require disulfide bond formation at the C‐(X)_11_‐C motif for import (Figure 6). However, significant decrease in rotenone‐sensitive NADH: duroquinone oxidoreductase activity and lower accumulation of the mature complex is observed (Figure 6). Therefore, we propose that the cysteines, while not strictly required for mitochondrial import, may have roles in protein folding and stability, assembly into the membrane arm, and assisting the ubiquinone reduction, and/or proton‐pumping capacity of complex I. The exact contribution of the C‐(X)_11_‐C motif to complex I activity and assembly still remains to be determined.

In summary, we have successfully used *Chlamydomonas* for testing the impact of human pathogenic nuclear mutations on complex I assembly/activity, revealing the utility of a unicellular plant model as an experimental system of study for unraveling the molecular basis of complex I deficiencies. Even accounting for variations in the assembly process between *Chlamydomonas* and human complex I (Subrahmanian et al., 2016), it is reasonable to expect that analyzing nuclear pathogenic mutations in *Chlamydomonas* will provide insight into their consequence on complex I function. Our work opens up new avenues of exploration through a systematic approach, where substitutions of all conserved residues in complex I subunits, individually and in concert, could be methodically employed to document the functional importance of each residue in complex I assembly and activity.

## CONFLICT OF INTEREST

The authors declare that they have no conflicts of interest with the contents of this article.

## AUTHOR CONTRIBUTIONS

NS and PH contributed to the conception and design of the study, the acquisition, analysis, interpretation of the data, and writing of the manuscript. ADC contributed to acquisition, analysis or interpretation of the data, and critical reading of the manuscript. TAF acquired data.
