# Supplementary material for: Chlamydomonas reinhardtii as a plant model system to study mitochondrial complex I dysfunction
Source: Plant Direct. 2020 Feb 3;4(2):e00200. doi: 10.1002/pld3.200 (PMC6996877; doi:10.1002/pld3.200)
Supplement: Supplementary file 1 [file PLD3-4-e00200-s001.pdf]

Chlamydomonas reinhardtii as a plant model system to study mitochondrial complex I dysfunction

Nitya Subrahmanian, Andrew David Castonguay, Thea Aspelund Fatnes, and Patrice Paul Hamel *.

Nitya Subrahmanian

Department of Molecular Genetics, The Ohio State University, Columbus, Ohio, USA.

Plant Cellular and Molecular Biology Graduate Program, The Ohio State University, Columbus, Ohio, USA.

Email: subrahmanian.1@buckeyemail.osu.edu

Andrew David Castonguay

Department of Molecular Genetics, The Ohio State University, Columbus, Ohio, USA.

Molecular Genetics Graduate Program, The Ohio State University, Columbus, Ohio, USA.

Email: castonguay.3@buckeyemail.osu.edu

Thea Aspelund Fatnes

Department of Molecular Genetics, The Ohio State University, Columbus, Ohio, USA.

Present address: Fürst Medical Laboratory, Soren Bulls Vei 25, 1051 Oslo, Norway.

Email: t.aspelund.f@gmail.com

Patrice Paul Hamel

Department of Molecular Genetics, Department of Biological Chemistry and Pharmacology, The Ohio State University, Columbus, Ohio, USA.

* To whom correspondence should be addressed: Patrice P. Hamel, Room 582 Aronoff Laboratory, 318 W. 12th Ave, Columbus, Ohio 43210, USA. Tel.: +1 614-292-3817; Fax: +1 614-292-6345;

E-mail: hamel.16@osu.edu.

List of Supplemental Information

Supplemental Methods:

Supplemental Method S1. Insertional mutagenesis and phenotypic screening of complex I mutants

Supplemental Method S2. Genetic analysis of the *amc* mutants

Supplemental Method S3. Ten-fold dilution series and growth curve analysis

Supplemental Method S4. Nucleic acid extraction, diagnostic PCRs and real-time quantitative PCRs

Supplemental Method S5. Plasmid construction

Supplemental Method S6. Complex II+III and Complex IV enzymatic assays

Supplemental Method S7. Immunoblotting analysis

Supplemental Figures:

Supplemental Figure S1. Additional Blue Native PAGE immunoblotting analyses.

Supplemental Figure S2. The *amc8* to *amc13* mutations are recessive.

Supplemental Figure S3. The *amc9* mutation is linked to the insertional cassette.

Supplemental Figure S4. The *NUO5* gene encoding the 24 kDa complex I subunit is disrupted in the *amc9* strain.

Supplemental Figure S5. Alignment of NUO5 / NDUFV2 / 24 kDa subunits.

Supplemental Figure S6. The FLAG-tagged variants of NUO5 are expressed in the *amc9* mutant.

Supplemental Figure S7. The wild-type *NUOB10* gene restores heterotrophic growth to an *amc5* mutant.

Supplemental Figure S8. Alignment of NUOB10 / NDUFB10 / PDSW subunits.

Supplemental Figure S9. The FLAG-tagged variants of NUOB10 are produced in the *amc5* mutant.

Supplemental Tables:

Supplemental Table S1. Sequence of primers used in this study.

Supplemental Table S2. Primers used for recombinant cloning.

Supplemental Table S3. Method of plasmid construction.

Supplemental Table S4. Summary of biolistic transformation.

Supplemental Methods

Supplemental Method S1: Insertional mutagenesis and phenotypic screening of complex I mutants

Transformation of $4C^-$ (*mt*$^-$; *arg7-8*) [CC-5590] strain was conducted after autolysine treatment by electroporation (Shimogawara *et al.*, 1998). The strain was grown in liquid TARG medium for 2-3 days until exponential growth (3 – 6 x 10^6 cells. mL^{-1}). For each transformation, 2.5 x 10^7 cells were electroporated at 1.3 kV and 10 µF with 100 ng of hygromycin B resistance cassette (iHyg3) and 20 µg of herring sperm DNA. The iHyg3 cassette consists of the *APHVII* gene with an *RBCS2* intron, under the control of the β2 tubulin-encoding gene (*TUB2*) promoter and the *RBCS2* terminator (Figure S4A). This cassette was amplified from the pHyg3 plasmid (Berthold *et al.*, 2002) using the primers: APH7-F and APH7-R (Table S1). The transformants were selected on TARG+HyB solid medium. Candidate complex I-deficient mutants were identified by scoring for the SID phenotype (Barbieri *et al.*, 2011). Hygromycin B resistant (HyB^R) colonies, which appeared after 10 days of incubation, were transferred into 400 µl of TARG + HyB liquid medium in 96-well plates, each well containing a single colony. The 96-well plate was incubated for five days at 25°C in continuous light at 50 µmol m^{-2} s^{-1}. The liquid cultures were then replica-plated onto two solid TARG media, incubated in the light and dark for five days. Transformants with a SID phenotype were selected and sub-cloned three times to obtain a single colony prior to further analysis. The *amc* mutant strains isolated in this study and their corresponding *Chlamydomonas Resource Center* reference numbers are as follows: *amc8 (1H5)* [CC-5600], *amc9 (41D9)* [CC-5601], *amc10 (12C)* [CC-5606], *amc11 (10G11)* [CC-5608], *amc12 (6E9)* [CC-5611], *amc13 (4C3)* [CC-5612], and *amc13(16)* [CC-5613].

Supplemental Method S2: Genetic analysis of the amc mutants

Genetic crosses were done as in (Harris, 1989) with some modifications. Vegetative cells were resuspended in TAP liquid medium lacking nitrogen (TAP-N), at 25°C in low light at .5-1 µmol m^{-2} s^{-1}, with shaking for a minimum of 5 hr. Equal volumes of the resultant gametes (of opposite mating types) were mixed and incubated in 50 µmol m^{-2} s^{-1} light at 25°C overnight to accomplish mating. In some cases, 10 mM dibutyryl-cAMP (WJ Pharmaceuticals, 100862) and/or 1 mM IBMX (Sigma, 15879-1G) were added to the mixture to stimulate mating. The mixture was plated on TAP-N solid medium (containing 3% (w/v) select agar) and incubated in high light at 25°C for 5 days, to allow the maturation of meiotic zygotes. Meiosis was induced by transferring the zygotes on TARG solid medium, in high light at 25°C,

for at least 12 – 16 hr. The meiotic progeny was obtained through bulk germination or tetrad dissection of zygotes.

In this study, the background strains 141 (*mt^+; arg9-2*), CC-125 (137C^+), or 1’ (*mt^+*) were crossed with the original *amc* mutants. Meiotic *amc* progeny derived from these crosses were also used for experimentation. Especially for *amc10* and *amc13*, their meiotic derivatives *amc10(12C)* and *amc13(16)* were used for analyses. For constructing *amc*/+ diploids, the *amc* mutants (*mt^-*; *amc*; *APHVII*; *arg7-8*) were crossed with CC-125 and the mating mixture was directly plated on selective medium (TAP + HyB). Individual diploids or meiotic progeny were subcloned to a single colony and their mating type was determined by diagnostic PCR (Werner & Mergenhagen, 1998).

Supplemental Method S3: Ten-fold dilution series and growth curve analysis

The SID phenotype was tested by ten-fold dilution series as follows. One loop of cells grown on solid TARG plates (for 3-5 days) was resuspended in 500 µl of liquid TARG medium. The cell density was measured spectrophotometrically at 750 nm and normalized to an OD_{750} = 2.0 by dilution. This normalized suspension was used as the starting material [1] for making five serial ten-fold dilutions [10^{-1}, 10^{-2}, 10^{-3}, 10^{-4}, and 10^{-5}]. A volume of 8 µl for each dilution was plated on solid TARG plates. For scoring the SID phenotype, two plates were prepared simultaneously and incubated at 25°C, one in continuous light and another in continuous dark, for at least 7 days. The light-incubated plate served as a control for confirming equal cell density amongst multiple strains.

To determine the generation time, liquid cultures were inoculated with a starting cell density of 10^5 cells. mL^{-1} in 50 mL TARG medium. Growth in liquid culture was observed by evaluating cell density at A_{750}. Measurements were taken every 8 hr over a period of 10 days. For each strain, three biological replicates were inoculated in continuous light at 25 µmol m^{-2} s^{-1} or in continuous darkness. Growth rate µ was calculated as $3.3*[\log_{10}N – \log_{10}N_0] / (t_N-t_0)$, where N is the final cell density at time t_N and N_0 is the initial cell density at time t_0. The generation time was calculated as 1/ µ (Harris, 1989, Kropat *et al.*, 2011).

The time periods used for calculating doubling times are as follows: For wild-type (4C^-) and [*amc9*; *NUO5*] strains in the light t_N = 86 hr and t_0 = 26 hr, and in the dark t_N = 110 hr and t_0 = 38 hr; for the *amc9* strain in the light t_N = 110 hr and t_0 = 38 hr, and in the dark t_N = 170 hr and t_0 = 86 hr; for wild-type (3A^+) in the light t_N = 96 hr and t_0 = 24 hr; for wild-type in the dark t_N = 120 hr and t_0 = 36 hr; for *amc5* in the

light t_N = 85 hr and t_0 = 37 hr; for *amc5* in the dark t_N = 123 hr and t_0 = 49 hr; for [*amc5*; *NUOB10*] in the light t_N = 85 hr and t_0 = 37 hr and for [*amc5*; *NUOB10*] in the dark t_N = 123 hr and t_0 = 37 hr.

Supplemental Method S4: Nucleic acid extraction, diagnostic PCRs and real-time qPCRs

Genomic DNA was extracted from *Chlamydomonas* by the phenol-chloroform method with some modifications (Sambrook *et al.*, 1989). One or two loops of cells, grown for two to three days in continuous light at 50 µmol m^{-2} s^{-1} on TARG solid medium, was harvested and resuspended in buffer (10 mM Tris-HCl, pH 8, 10 mM EDTA, 10 mM NaCl, 15% (w/v) glycerol). Cells were lysed by sonication for 5 s at 9 W output. Proteins were degraded by treatment with 100 µg proteinase K (Invitrogen, 25530049) and the extract was incubated at 55 ºC for one hour. RNA was degraded with 50 µg RNase A (Amresco, 0675-250mg). Nucleic acids were extracted twice by phenol-chloroform and DNA was precipitated by adding 2.2 volumes of ethanol and .1 volume of 3 M Na acetate pH 5.5.

The sequences of primers used for diagnostic PCR are shown in Table S1. For diagnostic PCR analysis, GoTaq Polymerase (Promega, M3008) was used as recommended by the manufacturer. To enhance the amplification of GC-rich regions, 2.5% (v/v) DMSO was used in each reaction in addition to a denaturation temperature of 98 ºC (instead of 95 ºC). For sequencing analyses, PCR products were gel-purified using the NucleoSpin Gel Extraction Kit (Machery Nagel, 740609.25) as per the manufacturer's instructions. The purified PCR product was then cloned into pGEM-T Easy Vector Systems (Promega, A1360) and then sequenced with T7 and SP6 primers flanking the cloning site.

For real-time quantitative PCR, RNA was prepared as in (Newman *et al.*, 1990). RNA was extracted from 2 x 10^8 cells grown in liquid culture. Nucleic acids were extracted twice with equal volume of phenol-chloroform (pH 5.0) and RNA was precipitated after overnight incubation at -20°C with 1/3rd volume of 8 M LiCl. Eight micrograms of RNA were treated with RQ1 RNase-free DNase I (Promega, M6101). Reverse transcription was achieved with 400 units of M-MLV Reverse transcriptase (Life Technologies, 28025-013) using one microgram of Random Hexamers (Promega, C1181), following the manufacturer's protocol. Amount of cDNA equivalent to 50 ng or 100 ng of total input RNA was used as template for qPCR using SensiMix (Bioline, QT-650-05) on a Mastercycler ep gradientS realplex thermocycler (Eppendorf). The sequences of primers used in qPCR are detailed in Table S1. The primer pairs NUO5E2L2 / NUO5E3R and NUOB10E4L / NUOB10E4R were used to amplify *NUO5* and *NUOB10* mRNA, respectively. The transcript abundance of the housekeeping genes *CBLP* (*Cre13.g599400*) and

EIF1A (*Cre02.g103550*) were determined using the primers CBLP-F / CBLP-R and EIF1A-Fw / EIF1A-Rev, respectively. Transcript abundance of E3 ubiquitin ligase *UBI* (*Cre03.g159200*) was determined by using primers Ubiupper / Ubilower and *TUA2* (*Cre04.g216850*) using Tub-F / Tub-R (de Montaigu *et al.*, 2011). Primer efficiencies for primers binding to *NUO5*, *NUOB10*, *TUA2*, *CBLP*, *UBI*, and *EIF1A*, determined by calibration curves, were 95%, 100%, 100%, 108%, 93%, and 99.8%, respectively. The target transcript was normalized to three reference transcripts by the Livak $2^{-\Delta\Delta Ct}$ method (Livak & Schmittgen, 2001). Three independent biological replicates were performed, each analyzed using two technical replicates. The qPCR reactions were denatured at 98 ºC for 15 s, annealed at 60 ºC for 20 s and extended at 72 ºC for 20 s. Relative fold change was determined by normalizing to the average of the isogenic wild-type strain. Results are represented as percentage of wild-type strain.

Supplemental Method S5: Plasmid construction

Plasmids expressing the *NUO5* and *NUOB10* wild-type and mutant genes were generated by recombination-based cloning in yeast. The pRS426-ble plasmid, containing three markers allowing for selection in algae, yeast, and bacteria, was used as the vector (Noor-Mohammadi *et al.*, 2014). It contains the *ble* gene conferring zeocin resistance for selection in *Chlamydomonas*, the *URA3* gene for selection in *S. cerevisiae,* and the *bla* gene conferring ampicillin resistance in *E. coli.* The pRS426-ble plasmid was linearized with *Not*I and *Ale*I (pB-NA). The *NUO5* and the *NUOB10* genes were cloned between the *Not*I and *Ale*I sites in pRS426-ble. The *NUO5* and *NUOB10* genes were expressed under their putative native promoters by including the entire intergenic region upstream of each gene (1220 bp and 1075 bp for *NUO5* and *NUOB10,* respectively). Each gene, including its promoter region, was amplified from purified genomic DNA using Velocity DNA Polymerase (Bioline, BIO21098) as consecutive overlapping fragments (Table S2). The sequence corresponding to the C-terminal FLAG-tag and site-directed mutations were introduced by PCR with appropriately designed primers (Table S2). The overlapping PCR fragments and linearized vector (pB-NA) were assembled via *in vivo* molecular recombination in yeast (Table S3). The *S. cerevisiae* strain CW04 (Banroques *et al.,* 1986) was used for recombination. Two hundred nanograms of each linear fragment was introduced into the strain by the one-step transformation method (Chen *et al.*, 1992, Saint-Georges *et al.*, 2002) and cells containing the recombinant clones were selected based on uracil prototrophy. Successful recombinants were identified based on diagnostic PCR and restriction digestion, verified by sequencing, and introduced into the respective *Chlamydomonas* strains by biolistics. However, the *ble* selection in *Chlamydomonas* was not successful as it led to the emergence of spontaneous zeocin-resistant colonies in which the transforming DNA was absent. Hence,

the *ble* selection marker was substituted with the *APHVII* selection marker (iHyg3) conferring HyB resistance (for the *NUOB10* clones) or *APHVIII* selection marker (iPm) conferring Pm resistance (for the *NUO5* clones). The *ble* marker (1.18 kb) was excised from each plasmid with *Eco*RI and *Not*I restriction enzymes. The iHyg3 and iPm markers were amplified from the plasmids pHyg3 and pSL18 (Berthold *et al.*, 2002, Depege *et al.*, 2003), respectively, using Velocity DNA Polymerase (Bioline, BIO21098) with primers including a 25 bp overlap with the digested vector (Table S2). Cloning of the new selection markers were achieved using the In-Fusion HD Cloning Kit (Clontech, 639648) according to the manufacturer's protocol (Table S3). The recombinant clones finally used for *Chlamydomonas* transformation are: pRS426-iPm-NUO5-WT-FLAG and pRS426-iPm-NUO5-K230R-FLAG for the *amc9* strain (41D9); pRS426-iHyg3-NUOB10-WT-FLAG, pRS426-iHyg3-NUOB10-C79S-FLAG, pRS426-iHyg3-NUOB10-C91S-FLAG, and pRS426-iHyg3-NUOB10-C79SC91S-FLAG for the *amc5* strain (87D3) (as detailed in Tables S3 and S4).

Supplemental Method S6: Complex II+III and Complex IV enzymatic assays

Activity assays were conducted on crude membrane extracts that were prepared as described in Materials and Methods section. Complex II+III activity assay was conducted in the presence of 20.25 mM succinate (Acros organics, 158751000), 1 mM KCN (FisherScientific, P223I-100) and 56 µM equine heart cytochrome *c* (Sigma, 2506-500mg). The activity was determined as the rate of cytochrome *c* reduction and measured spectrophotometrically at $A_{550.}$ Complex II+III activity was calculated using molar extinction coefficient for cytochrome *c* at $\Delta\varepsilon_{550nm} = 19.6$ mM^{-1} cm^{-1}, in the absence and presence of complex III-specific inhibitor myxothiazol (3 µM) (Sigma, T5580). Complex IV activity assay was conducted in the presence of 1% Triton X-100 and 56 µM reduced cytochrome *c*. Cytochrome *c* was reduced with two times the amount of sodium dithionite and purified with a PD10-desalting column with Sephadex G-25 resin (GE Lifesciences, 17085101) according to the manufacturer's protocol. Complex IV activity was calculated using molar extinction coefficient for cytochrome *c* at $\Delta\varepsilon_{550nm} = 19.6$ mM^{-1} cm^{-1}, in the absence and presence of complex IV inhibitor KCN (1 mM).

Supplemental Method S7: Immunoblotting analysis

BN-PAGE was completed as described in Materials and Methods section. For SDS-PAGE, 10 µg of crude membrane proteins was separated by 12.5% acrylamide gel and immunoblotting was performed according to established protocols (Sambrook *et al.*, 1989). The separated proteins were electro-blotted onto PVDF

membranes and custom-made rabbit polyclonal antibodies specific for *Chlamydomonas* complex I subunits (from Genescript, as described in (Barbieri *et al.*, 2011)), were used. Membranes with electroblotted proteins separated in BN-PAGE gels were probed overnight at 4°C with 1:3000 diluted α-51 kDa, a polyclonal antibody that detects the soluble arm 51 kDa subunit. Membranes containing proteins resolved by SDS-PAGE immunoblots were probed at room temperature for 3 hr with 1:3000 diluted α-51 kDa, 1: 3000 diluted α-49 kDa, 1:2000 diluted α-TYKY and for 1 hr with 1:12,000 diluted α-cytochrome *f* (Dreyfuss et al., 2003). For rabbit polyclonal antibodies, a HRP-conjugated anti-rabbit Goat IgG (Biorad, 170-6515) at 1:10,000 dilution was used as the secondary antibody. For detecting the FLAG-tagged proteins, membranes were incubated with monoclonal anti-FLAG antibody (Sigma, F1804) at 1:5000 dilution overnight at 4°C followed by a HRP-conjugated anti-mouse Goat IgG (Pierce 18584_3) at a dilution of 1:2,500 as the secondary antibody. Intensity of bands in immunoblots were quantified using ImageJ (Schneider *et al.*, 2012).

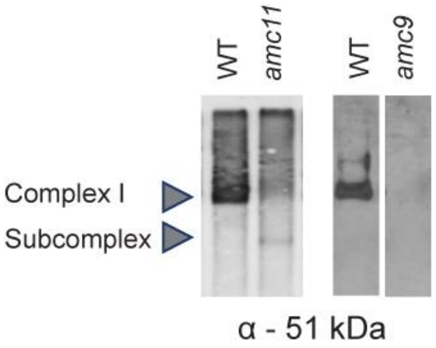

Supplemental Figure S1. Additional Blue-Native PAGE immunoblotting analyses.

Blue-Native PAGE followed by immunoblotting was conducted on crude membrane extracts using a polyclonal antibody to detect the 51 kDa subunit of complex I. The white vertical line between the lanes of WT and *amc9* denotes the assembly of lanes from the same blot.

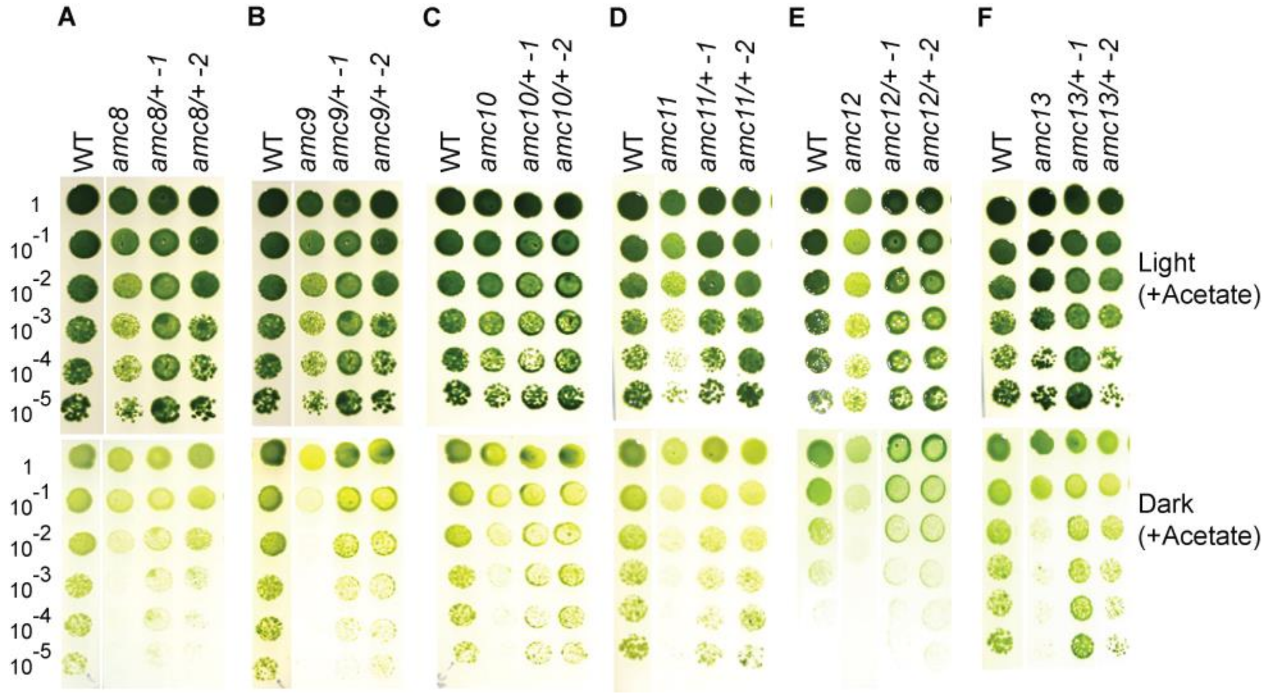

Supplemental Figure S2. The *amc8* to *amc13* mutations are recessive.

To test if the *amc* mutations causing the complex I-deficient phenotype are recessive or dominant with respect to the wild-type allele, heterozygous diploids (*amc*/+) were constructed by crossing *amc* mutants with a wild-type strain as described in Method S2. Two independent diploids from each cross were tested for growth in the dark by ten-fold dilution series. The dilution series were plated on acetate-containing medium and incubated in the light or dark for 10 days. The heterozygous *amc/*+ diploids were restored for growth in the dark, thereby indicating that all the *amc* mutations are recessive. White vertical lines indicate pictures of strains grown from the same plate and assembled for the display in the figure. The growth of the following diploids is depicted in panels A-F wherein WT (4C$^-$) and the respective *amc* mutants, used for constructing the diploids, were used as controls: A, *amc8/*+; B, *amc9/+*; C, *amc10/*+; D, *amc11/*+; E, *amc12/*+; F, *amc13/*+.

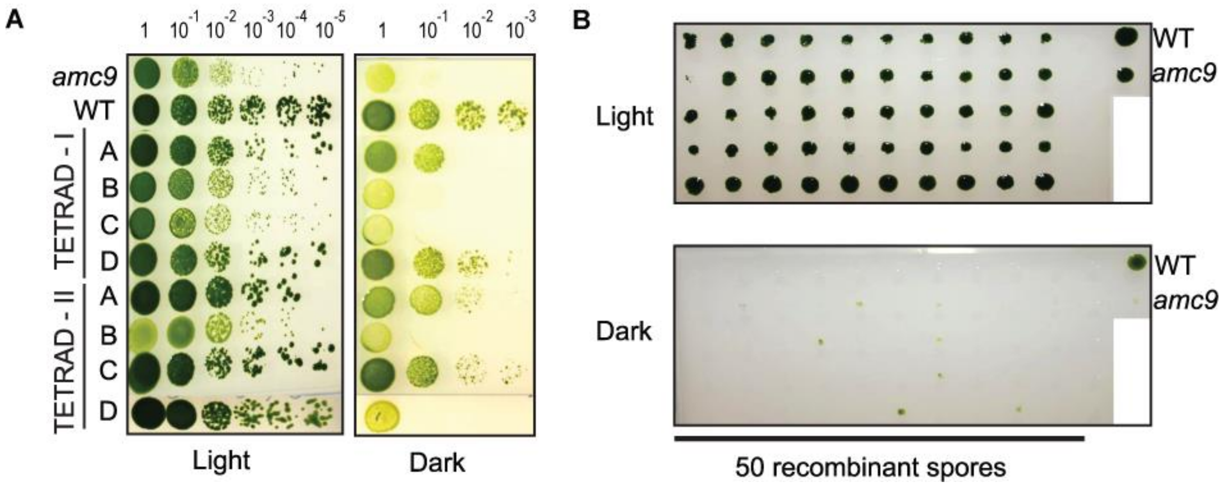

Supplemental Figure S3. The *amc9* mutation is linked to the insertional cassette.

Meiotic zygotes were obtained by crossing the complex I proficient strain 141 (*mt*$^{+}$; *arg9*-2) with the complex I-deficient *amc9* (*3*$^{-}$) strain (*mt*$^{-}$; *nuo5::APHVII*). The meiotic zygotes, obtained from the same genetic cross, were used for both tetrad dissection (A) and bulk germination (B). (A) The growth phenotype of two out of seven tetrads dissected from the above-mentioned cross is shown here by ten-fold dilution series. The dilutions were plated on acetate-containing medium and incubated in the light and in the dark for 15 days. The WT strain and the *amc9* strain shown here are the original parental strains. (B) Fifty recombinant hygromycin B resistant progeny, were analyzed from bulk germination of meiotic zygotes. The progeny were replica-plated on TARG solid medium in the light or dark and the growth phenotype was scored after 15 days of incubation. The WT and *amc9* strains shown here are 4C^{-} and *amc9 (41D9)*, respectively. Out of 50 antibiotic-resistant meiotic progeny that were tested, all of them displayed a SID phenotype confirming that the *amc9* mutation is tightly linked to the insertional cassette.

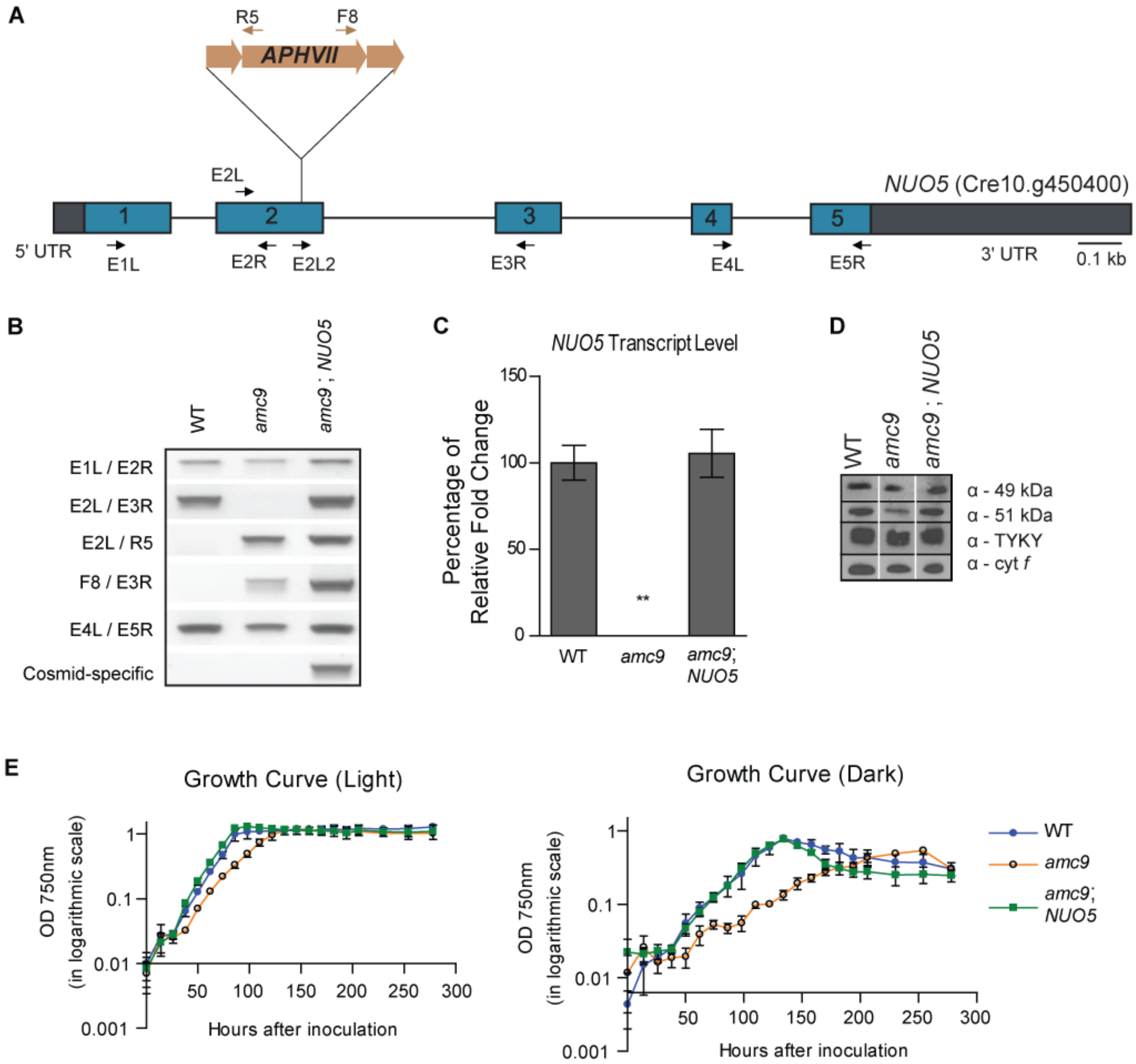

Supplemental Figure S4. The *NUO5* gene encoding the 24 kDa complex I subunit is disrupted in the *amc9* strain.

(A) A diagram of the *NUO5* gene with the approximate position of the insertional cassette in the *amc9* mutant is depicted here. The brown arrows indicate primers APH7R5 and APH7F8 that bind to the *APHVII* gene in the iHyg3 cassette. The black arrows represent *NUO5*-specific primers NUO5E1L, NUO5E2R, NUO5E2L, NUO5E2L2, NUO5E3R, NUO5E4L, and NUO5E5R (Table S1). (B) Diagnostic PCR of the *NUO5* gene in wild-type (WT, 4C^{-}), *amc9 (41D9)*, and one *amc9* strain transformed with a cosmid containing the *NUO5* gene [*amc9*; *NUO5*] is shown here. The positions of the primers used for diagnostic PCR of the *NUO5* gene are represented in (A). The location of the insertion was confirmed by amplification of the genomic region spanning the insertion site using primer pairs NUO5E2L / NUO5E3R.

A band of expected size (715 bp) was amplified in the wild-type and not in the *amc9* mutant. The insertion of the full-length iHyg3 cassette was confirmed by amplifying across the *NUO5/iHyg3* junctions in the *amc9* mutant. Primer pairs amplifying from the 5′-end (NUO5E2L / APH7-R5) and from the 3′-end (APH7-F8 / NUO5E3R) of the iHyg3 insertional cassette yielded products of expected size, 524 bp and 955 bp, respectively, in *amc9* but not in the wild-type. Diagnostic PCRs conducted upstream and downstream of the insertion site yielded amplicons of the expected size, indicating the absence of other major rearrangements in the *NUO5* gene of the *amc9* strain. Diagnostic PCR of the [*amc9; NUO5*] transformant revealed the presence of the wild-type *NUO5* gene, in addition to the endogenous disrupted *NUO5* gene. The primers (PH3 and 9A2 PH3, Table S1) were used for determining the presence of the cosmid (last row), harboring the wild-type *NUO5* gene. (C) Real-time quantitative PCR was used to assess the quantity of *NUO5* mRNA relative to the transcripts of three reference genes *TUA2, CBLP* and *UBI.* The average was obtained from three biological replicates, each including two technical replicates. The error bars represent standard deviation of the mean. The results are represented as percentage of fold change relative to WT (WT set to 100). Statistically significant difference with respect to the wild-type was determined by two-tailed unequal variances *t*-test. ** indicates $p < .01$. (D) SDS-PAGE immunoblotting was conducted on 10 µg of partially purified membranes using polyclonal antibodies to detect complex I soluble arm subunits: α- 49 kDa, α- 51 kDa, α- TYKY. α– cyt *f* was used to confirm equal loading. Vertical white lines indicate the assembly of different lanes from the same immunoblot. (E) The growth of WT ($4C^-$), *amc9* (41D9) and [*amc9*; *NUO5*] was recorded by measuring optical density at 750 nm, in the light (22 µmol m^{-2} s^{-1}) or in the dark, over a period of 10 days. The average of three biological replicates is reported here, with error bars indicating standard deviation of the mean.

```
                          10         20         30         40         50         60         70
                  ....|....| ....|....| ....|....| ....|....| ....|....| ....|....| ....|....|
T.thermophilus   1 ---------- ---------- ---------- ---------- ---------- ---------- ----------
E.coli           1 ---------- ---------- ---------- ---------- ---------- ---------- ---mhen--q
Y.lipolytica     1 -------ml- -rlirprl-- ---------- ---------a alarpttrap --qalnarth ivsvhrnten
N.crassa         1 matkltpflm rtavraat-- ---------- ----rlstkp stiapvsrac lsisarrpsd tlmvhrntpd
D.melanogaster   1 -------mlt ncas------ ---------- -----k-tla avraniraia --tssarasd nlfvhrdtpe
D.rerio          1 -------mfl sstlrsav-- ---------- ---------s ytarqvrslh qtsaraga-g gifvhrdtpe
H.sapiens        1 -------mff saalrara-- ---------- ----ag-lta hwgrhvrnlh ktvmqngagg alfvhrdtpe
B.taurus         1 -------mfl saalrara-- ---------- ----ag-laa hwgkhirnlh ktavqngagg alfvhrdtpe
M.musculus       1 --------mf slalrara-- ---------- ----tg-laa qwgrharnlh ktavhngagg alfvhrdtpe
C.reinhardtii    1 ---------- -mlsrallla grlaatgqqq aastssravq plgsllqr-- -cnfatnstd ifnihkdtpe
P.patens         1 ------mrsw rvatralavi rn--aqgvra ppsaaapqla pfvpqiq--r revpsvnyst atfahvntpd
A.thaliana       1 -------mla rlaakrll-- ---------- ---------- -eirqvfrqp tsqvtrslst alnyhldspd
V.vinifera       1 -------mla rlatkrll-- ---------- ---------- -evrqifrqn h-qtsrsfst alnyhldspd

                               80         90        100        110        120        130        140
                  ....|....| ....|....| ....|....| ....|....| ....|....| ....|....| ....|....|
T.thermophilus   1 ------mgff ddkqdfleet fakyppegrr aaimpllrrv qqee-gwirp erieeiarlv gttptevmgv
E.coli           6 qpqteafels aaereaiehe mhhyedp--r aasiealkiv qkqr-gwvpd gaihaiadvl gipasdvegv
Y.lipolytica    39 nnpsipfefs penmkraeev iakyppqykk aavmplldig qrql-gytsi svmnyvakll emppmrvyev
N.crassa        55 nnpdipfkfs adnekvieei ikryppqykk aavmplldlg qrqh-gfcsi svmnevarll emppmrvyev
D.melanogaster  40 dnpnipfeft aenkkrveai lsiypeghkr gamipllдla qrqy-gwlpi samhkvaeil qlpnmrvyev
D.rerio         42 nnpdtpfeft penmkrveai innypeghka aatipvldla qrqn-gwlpi samnkvaevl giapmrvyev
H.sapiens       47 nnpdtpfdft penykrieai vknypeghka aavlpvldla qrqn-gwlpi samnkvaevl qvppmrvyev
B.taurus        47 nnpetpfdft penykrieai vknypeghka aavlpvldla qrqn-gwlpi samnkvaeil qvppmrvyev
M.musculus      46 nnpdtpfdft penykrieai vknypeghqa aavlpvldla qrqn-gwlpi samnkvaevl qvppmrvyev
C.reinhardtii   57 nnaatsfefs eatlkvvndi iaryppnykq saiipvldvt qqenggwlsl aamnrvakll dmapirvyev
P.patens        61 nnpdlkwdft panmekvkel lshypknykq savipmldla qqqnggwlsv qamnriaeiv dyapirvyev
A.thaliana      41 nkpdlpwefs eanqskvkei lsyypsnykq savipllдla qqqnggwlpv samnavakvi evapirvyev
V.vinifera      40 nnpnipwefn dankgkvkei lshypsnykq savipllдla qqqhggwlpv samdavakvv evapirvyev

                              150        160        170        180        190        200        210
                  ....|....| ....|....| ....|....| ....|....| ....|....| ....|....| ....|....|
T.thermophilus  64 asfysyyqfv ptgkyhlqvc atlsckl--a gaeelwdylt etlgigpgev tpdglfsvqk veclgschta
E.coli          73 atfysqifrq pvgrhviryc dsvvchi--n gyqgiqaale kklnikpgqt tfdgrftllp tcclgncdkg
Y.lipolytica   108 atfytmynrt pmgryhlqic tttpcql--c gsdgimeavq ntlnikpget tkdnlftlse veclgacvna
N.crassa       124 asfytmynrt pvgkfhvqac tttpcqlggc gsdvivkaik ehlgikqget tpdglftfie veclgacana
D.melanogaster 109 atfytmfmrk ptgkyhiqvc tttpcwl--r gsddiletck kqlgigvgdt tkdrkftise veclgacvna
D.rerio        111 atfytmflrq pvgkyhiqic tttpcml--c dsdsileaiq nklgikvget tadklftlte veclgacvna
H.sapiens      116 atfytmynrk pvgkyhiqvc tttpcml--r nsdsileaiq kklgikvget tpdklftlie veclgacvna
B.taurus       116 atfytmynrk pvgkyhiqvc tttpcml--r nsdsileaiq kklgikvget tpdklftlie veclgacvna
M.musculus     115 atfytmynrk pvgkyhiqvc tttpcml--r dsdsiletlq rklgikvget tpdklftlie veclgacvna
C.reinhardtii  127 atfytmfnrt kigkyhvqic gttpcrl--q gsqkieeait khlgigigqt tqdglftlge mecmgacvna
P.patens       131 atfysmfnrq pvgkyhllvc gttpcml--r gsrdiedall khlhvarnev tkdglfsvge mecmgccvna
A.thaliana     111 atfysmfnra kvgkyhllvc gttpcmi--r gsrdiesall dhlgvkrgev tkdglfsvge mecmgccvna
V.vinifera     110 atfysmfnra kvgkyhllvc gttpcmi--r gsreiedall khlgvkrnev tkdglfsvge mecmgccvna

                              220        230        240        250        260        270        280
                  ....|....| ....|....| ....|....| ....|....| ....|....| ....|....| ....|....|
T.thermophilus 132 pviqvnde-- -------pyv ecvtrarlea llaglragkr -leeielpgk cgh------- ----------
E.coli         141 pnmmide--- -------dth ahltpeaipe llerykk---- ---------- ---------- ----------
Y.lipolytica   176 pmmaind--- -------dyy edltpegtvk lledcka--- --gkmptpgp enhvr----- -----rdcep
N.crassa       194 pmvqind--- -------dyf edltpetikq vlsalkesvt dvskapqpgp qsg-r----- -----qtcen
D.melanogaster 177 pmvaind--- -------dyy edltskdmqd ilndlka--- --dkisppgp rng-r----- -----fasep
D.rerio        179 pmvqind--- -------nyy edlkpsdmeq iidelka--- --grvpppgp rsg-r----- -----fscep
H.sapiens      184 pmvqind--- -------nyy edltakdiee iidelka--- --gkipkpgp rsg-r----- -----fscep
B.taurus       184 pmvqind--- -------nyy edltpkdiee iidelka--- --gkipkpgp rsg-r----- -----fscep
M.musculus     183 pmvqind--- -------nyy edltpkdiee iidelka--- --gkvpkpgp rsg-r----- -----fccep
C.reinhardtii  195 pmvaiadytk gvsgfeyiyy edltpkdivn ildtikk--- --ggkpkpgs qyrlkaepag avhggekwvp
P.patens       199 pmivvadysn gvegysynyy edltpervve lveelkq--- --gkkpkwgt qhpkr----- -----incgp
A.thaliana     179 pmitvadysn gsegytynyf edvtpekvve iveklrk--- --gekpphgt qnpkr----- -----ikcgp
V.vinifera     178 pmitvadyst gsegytynyy edvtpkrvve ivemlrr--- --gekpphgt qnpqr----- -----trcgp
```

```
                         290       300
                     ....|....| ....|....| ....
T.thermophilus   175 hvhevev--- ---------- ----
E.coli           166 ---------- ---------- ----
Y.lipolytica     221 asgqkvllsk ephnvadflq egi-
N.crassa         243 aagltslts- epygpevtrs dl--
D.melanogaster   221 kgeptslse- epkgpgfglq agl-
D.rerio          223 aggltslte- pppgpgvgvr adl-
H.sapiens        228 aggltslte- ppkgpgfgvq agl-
B.taurus         228 aggltslte- ppkgpgfgvq agl-
M.musculus       227 aggltslte- ppkgpgfgvq agl-
C.reinhardtii    260 kdgettltg- aprapycrdl nata
P.patens         254 aggtttlls- epqappcrdl dac-
A.thaliana       234 eggnktllg- epkppqfrdl dac-
V.vinifera       233 eggnttllg- epkappcrdl dac-
```

Supplemental Figure S5. Alignment of NUO5 / NDUFV2 / 24 kDa subunit orthologs.

NUO5 / NDUFV2 / 24 kDa subunit proteins were aligned using Clustal Omega (BLOSUM 62 scoring matrix) and Bioedit (Sievers *et al.*, 2011, Hall, 1999). The conserved cysteines that coordinate the 2Fe-2S (N1a) cluster in NUO5 are highlighted in green. In *E. coli,* the N1a cluster has a high midpoint redox potential implying a possible role in electron transfer through a flavosemiquinone intermediate (Verkhovskaya *et al.*, 2008). However, in mammalian complex I this cluster exhibits a low midpoint redox potential implying inability for electron relay and hence NDUFV2 is alternatively proposed to function in the structural stability of the enzyme (Birrell *et al.*, 2013, Verkhovskaya *et al.*, 2008). The two cysteines that provide increased stability by forming a disulfide in *T. thermophilus* ortholog are highlighted in magenta (Sazanov & Hinchliffe, 2006). The conserved lysine residue that is mutated to arginine in the Parkinson's disease patients (Nishioka *et al.*, 2010) is highlighted in yellow. NCBI accession numbers are as follows: *T. thermophilus* (AAA97942), *E. coli* (EDV69206), *Y. lipolytica* (XP_502254), *N. crassa* (XP_961535), *D. melanogaster* (NP_573228), *D. rerio* (NP_957041), *H. sapiens* (NP_066552), *B. taurus* (NP_776990), *M. musculus* (NP_082664), *C. reinhardtii* (XP_001698508), *P. patens* (XP_001759238), *A. thaliana* (NP_567244), *V. vinifera* (XP_002281655).

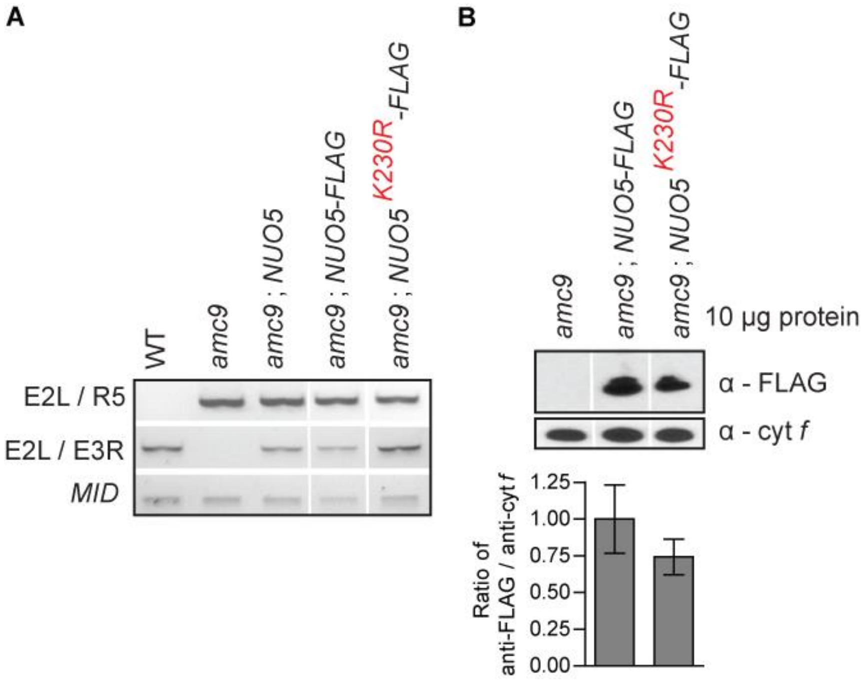

Supplemental Figure S6. The FLAG-tagged variants of NUO5 are expressed in the *amc9* mutant.

The *amc9* mutant was transformed with two constructs containing *NUO5* genomic DNA: i) wild-type *NUO5* sequence (*NUO5-FLAG*), or ii) mutant *NUO5* sequence encoding the K230R substitution (*NUO5*^{K230R}*-FLAG*). The wild-type (WT, 4C^{-}), *amc9* (*41D9*) mutant and [*amc9*; *NUO5*] strains were used as controls. White vertical thin lines indicate lanes that have been assembled together from the same gel. (A) Diagnostic PCR of the *NUO5* gene in the *amc9* transformants, at the site of insertion (NUO5 E2L / NUO5 E3R), resulted in amplification of wild-type sequence indicating the presence of transforming DNA. All transformants also contained the *amc9* insertional mutation (NUO5 E2L / APH7R5). The sequence and position of the primers is available in Table S1 and Figure S3. Amplification of the *MID* gene was used as control. (B) Immunoblotting was conducted on whole cell proteins separated by SDS-PAGE using monoclonal antibodies to detect FLAG tag (α- FLAG). Polyclonal antibody α– cyt *f* was used to test for equal loading. The proteins were quantified with ImageJ software from three independent biological replicates. No significant difference in NUO5-FLAG accumulation was observed for the wild-type NUO5-FLAG and the K230R NUO5-FLAG variant.

```
                              10         20         30         40         50         60         70
                      ....|....| ....|....| ....|....| ....|....| ....|....| ....|....| ....|....|
C.reinhardtii         ---------- ---------- -msasveerq sfhdkvndvf knltge---- -------agk -rwarpdepi  37
H.sapiens             mpdswdkdvy pepprrtpv- -----qpnpi vymmkafdli vdrpvtlvre fierqhaknr yyyyhrqyrr  64
B.taurus              mpdswdkdvy pepprrtpap spqtslpnpi tyltkafdll vdrpvtlvre fierqhaknk yyyyhrefrr  70
M.musculus            mpdswdkdvy peppsrtpap spqtslpnpi tyltkaydlv vdwpvtlvre fierqhaknr tyyyhrqyrr  70
G.gallus              mpddpdlevy ktppsrtpvt estsalpnpv tfiqtvfnyv idapvtfvre wierqqaknk syyyhqkfrr  70
X.tropicalis          mpdewskday peppsrtpap dkhtalpnpa vivsklfyya vdvpvtqfhd wverqrsrnt yyyyhrqyrr  70
D.rerio               mpqdynkday peppsrtpme nkqtavpnpa vlitkvfyyt vdlpvstfrg iverfrgdkk ayyyhqkfrr  70
D.melanogaster        ---------- -mpeprs--- --------pm asfaesvlnv idgpitwfre sivepn-qqk qnwyhqrfrr  47
A.thaliana            ---------- ---------- ---------- ---------- --mgr----- --------kk -glpefeesa  14
V.vinifera            ---------- ---------- ---------- ---------- --mgr----- --------kk -gtvefdesp  14
P.patens              ---------- ---------- ---------- ---------- --mvf----- --------ns -dypkfdqea  14
N.crassa              ---------- ---------- ---------- ---------- --mptp---- -------esa -aflakkptv  16
Y.lipolytica          ---------- ---------- ---------- ---------- ---------- ---------- --msehqrpe  8

                              80         90        100        110        120        130        140
                      ....|....| ....|....| ....|....| ....|....| ....|....| ....|....| ....|....|
C.reinhardtii         lhtln--dek eephiffhnt nyrtsrkyin vektkilreq alecvraagt aryhkctevm krlqaavrva  105
H.sapiens             vpdit-ecke edimcmyeae mqwkrdykvd qeiinimqdr lkacqqregq nyqqncikev eqftqvakay  133
B.taurus              vpdit-ecqe kdvlcmfeae mqwrrdykvd qeivniiqer lkacqqrege shrqncakel eqftqvvkay  139
M.musculus            vpdit-ecke gdvlciyeae mqwrrdfkvd qeimniiqer lkacqqrege nyqqncakel eqftkvtkay  139
G.gallus              vpdvs-ecle gdylcfyeae aqwrrdrlvd qqiveivrer lgackqregp nqfqncakea eqlaqvtkay  139
X.tropicalis          vpdlt-ecev gdylcyfeae mqwkrdhkvd qeivkilqer mracqqregh syvqncakev aqfteaakgy  139
D.rerio               vpelt-qcqq gdflcyyeae mqwrrdykvd qeivkvmqnr lkacqqregh syvqncqkei kqfketsqaf  139
D.melanogaster        vptid-qcyt ddavcrfead qqfrrdrmvd neivnilrqr fedctlyeap dhmvkcrplm dqyekatenw  116
A.thaliana            pdgfdpenpy kdpvamvemr ehivrekwiq iekakilrek vkwcyrvegv nhyqkcrhlv qqyldstrg-  83
V.vinifera            pdgydpenpy kdpvvyfdmr eyqvrekwid iekakilrek lkwcyriegv nhlqkcrhlv qqyldatrg-  83
P.patens              pddfdpakpy adpvaffeqr efvvrekliq vekakilrek lrqcywkegv nhyqkcrplv qkymascqn-  83
N.crassa              pptfdg-vdy ndtkrlkqaq daiireqwvr vmmgrlvree lskcyyregv nhlekcghlr erylqlhsen  85
Y.lipolytica          lvsfdd-iny ndhkkvreaq esytreqfir lealktvrka lekcyeesgp nhfedcknla eqyldmlpth  77

                             150        160        170        180        190
                      ....|....| ....|....| ....|....| ....|....| ....|....| ..
C.reinhardtii         snvdrgplar krdvgfiy-- hnnrlrelqq qaaeleienp fpapakqatg gy 155
H.sapiens             qd-------r yqdlgayssa rkclakqrqr mlqerkaak- eaaaats--- -- 172
B.taurus              qd-------r yhdlgahysa rkclakqkqr mlaerkaak- eaaaa----- -- 176
M.musculus            qd-------r yldlgayysa rkclakqkqr mleerkaar- qeaaa----- -- 176
G.gallus              qe-------r ygdlgvhgna rtclmkqkhr mieemkaqe- nasq------ -- 175
X.tropicalis          qs-------r ygdlgaygna rkclmkqkhr miaerkaaa- qaq------- -- 174
D.rerio               qs-------r ygdlgaygsa rkclmkqker mmkeaqke-- ---------- -- 170
D.melanogaster        fi-------k ygdlggyana ktaymkqkhr liwerrhgp- vgsgmkeeaa h- 159
A.thaliana            ---------- ---vgwgkdh rpislhgpkp eaveae---- ---------- -- 106
V.vinifera            ---------- ---igwgkdg rhpslhgpkv eaese----- ---------- -- 105
P.patens              ---------- ---igwgkda rpsylnhl-- ---------- ---------- -- 98
N.crassa              rv-------- ---qgylfeq qnhfanqpkq ---------- ---------- -- 104
Y.lipolytica          rl-------- ---qgylgyq rndpsk---- ---------- ---------- -- 92
```

Supplemental Figure S7. Alignment of NUOB10 / NDUFB10 / PDSW subunits orthologs. NUOB10 / NDUFB10 / PDSW subunit proteins were aligned using Clustal Omega (BLOSUM 62 scoring matrix) and Bioedit (Sievers *et al.*, 2011, Hall, 1999). The conserved PDSW sequence at the N-terminus is highlighted in green. The two cysteines of the C-$(X)_{11}$-C motif are highlighted in yellow, the C-$(X)_{6}$-C motif in pink and the fifth cysteine in the human ortholog is highlighted in blue. NCBI accession numbers are as follows: *D. melanogaster* (NP_651972), *G. gallus* (XP_414844), *H. sapiens* (O96000), *B. taurus* (Q02373), *M. musculus* (Q9DCS9), *X. tropicalis* (NP_001017250), *D. rerio* (Q6PC16), *C. reinhardtii* (XP_001694041), *P. patens* (XP_001770976), *A. thaliana* (NP_566608), *V. vinifera* (XP_002276810), *N. crassa* (CAA48768), *Y. lipolytica* (XP_002142991).

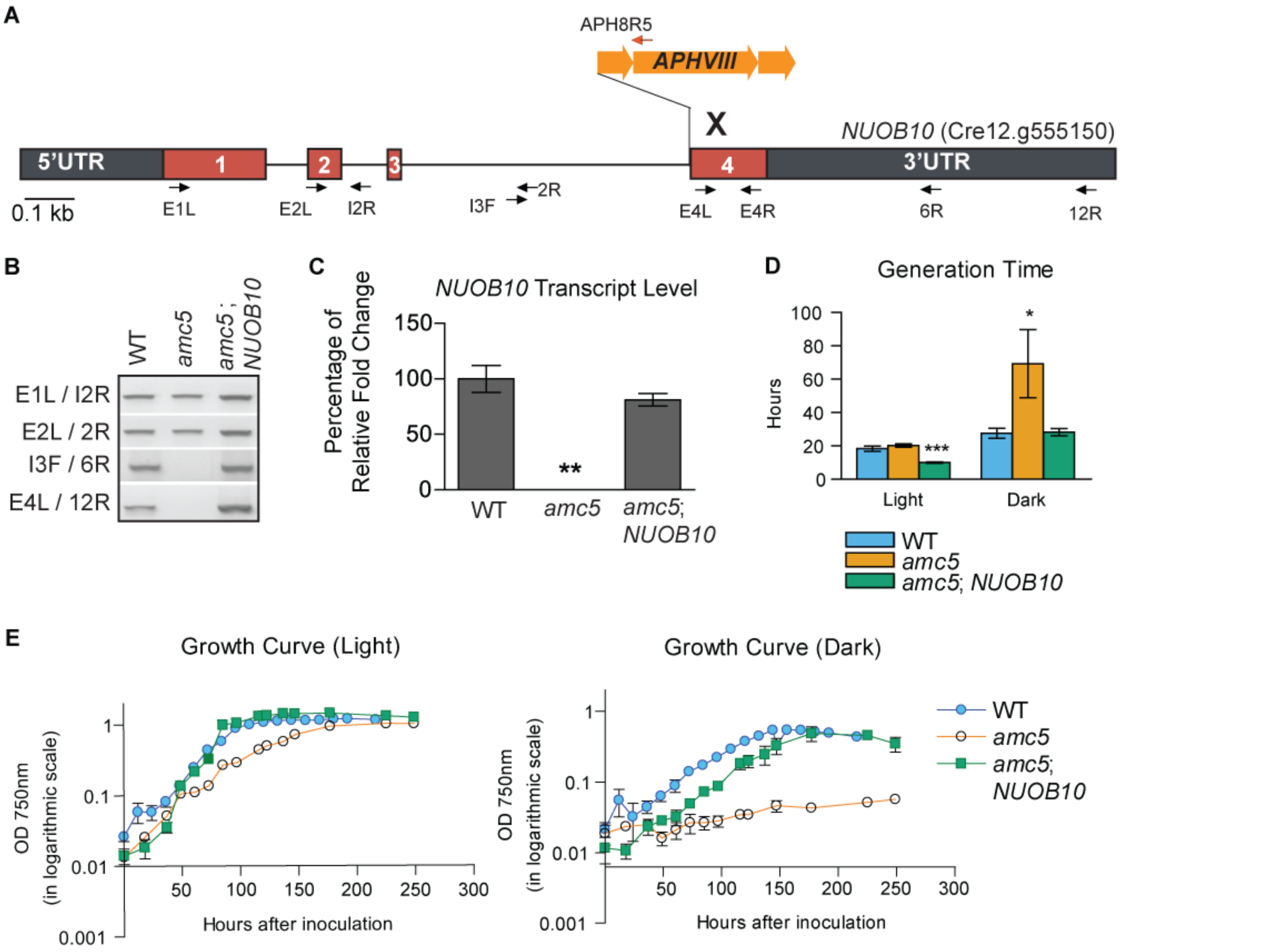

Supplemental Figure S8. The wild-type *NUOB10* gene restores heterotrophic growth to the *amc5* mutant.

(A) A diagram of the *NUOB10* gene with the position of the insertional cassette in the *amc5* mutant is depicted here. The gray and red rectangles represent UTRs and the coding sequence of *NUOB10*, respectively. The introns are denoted by thin black lines. The *X* mark indicates deletion of the *NUOB10* genomic region downstream of the insertion site. The black arrows indicate the primer binding sites for the *NUOB10*-specific primers and the orange primer is specific to the *APHVIII* insertional cassette conferring paromomycin resistance (iPm). (B) Diagnostic PCR of the *NUOB10* gene in the *amc5* strain reveals the presence of the molecular lesion as previously described in (Barbieri *et al.*, 2011). The [*amc5*; *NUOB10*] strain contains an intact copy of the *NUOB10* gene as expected. (C) Real - time RT-qPCR was used to assess the quantity of *NUOB10* mRNA relative to three reference genes *TUA2*, *CBLP* and *EIFA*.

The average was obtained from three biological replicates, each including two technical replicates. The error bars represent standard deviation of the mean. The results are represented as percentage of fold change relative to WT (WT set to 100). Statistical significance was determined with respect to wild-type by two-tailed unequal variance *t*-test. ** indicates *p*-value < .01. All primer sequences described in (A – C) are detailed in Supplementary Table S1. (D and E) The growth of the wild-type (WT, $3A^+$), *amc5* and [*amc5*; *NUOB10*] strains was recorded by measuring optical density at 750 nm, in the light or in the dark, over a period of 10 days. The average of three biological replicates is reported in (E), with error bars indicating standard deviation of the mean. The average generation time for each strain calculated from growth curves is depicted in (D). The error bars represent standard deviation of the mean. Statistical significance was determined by two-tailed equal variances *t*-test and * indicates *p*-value < .05, *** indicates *p*-value < .001.

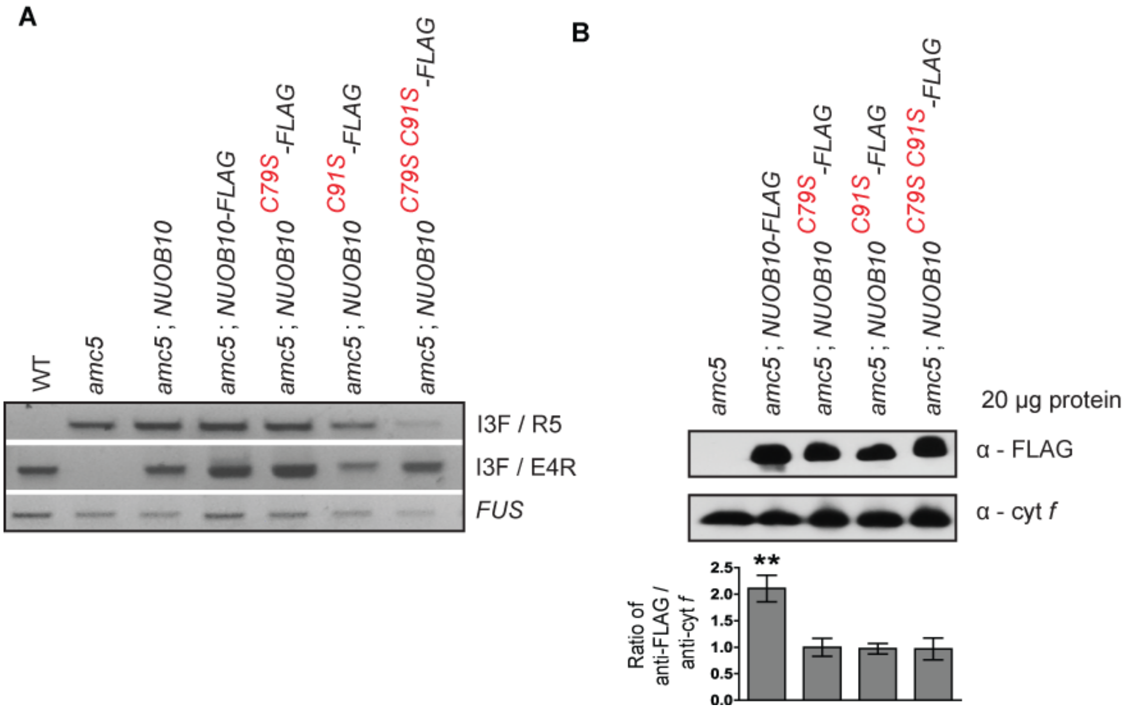

Supplemental Figure S9. The FLAG-tagged variants of NUOB10 are produced in the *amc5* mutant.

The *amc5* mutant was transformed with four constructs containing the *NUOB10* genomic DNA: i) wild-type *NUOB10* sequence (*NUOB10* or *NUOB10-FLAG*), and mutant *NUOB10* sequences encoding the variants with ii) the C79S substitution ($NUOB10^{C79S}$-*FLAG*), iii) the C91S substitution ($NUOB10^{C91S}$-*FLAG*), or iv) the C79S and C91S double substitutions ($NUOB10^{C79SC91S}$-*FLAG*). The wild-type (WT, $3A^{+}$), *amc5* mutant and [*amc5*; *NUOB10*] strains were used as controls. (A) Diagnostic PCR of the *NUOB10* gene in the *amc5* transformants, at the site of insertion (NUOB10 I3F / NUOB10 E4R), shows amplification of wild-type sequence indicating the presence of transforming DNA. All transformants also contain the *amc5* insertional mutation (NUOB10 I3F/ APH8R5). The position of the primers is available in Figure S8A. Amplification of the *FUS* gene was used as control. (B) *(top):* SDS-PAGE immunoblotting was conducted on whole cell protein extract using monoclonal α- FLAG antibody to detect FLAG-tagged NUOB10. Polyclonal antibody to detect α– cyt *f* was used to test for equal loading. *(below):*The immunoblots were quantified by ImageJ software from three independent biological replicates (Schneider *et al.*, 2012). The transformant [*amc5*; *NUOB10-FLAG*] accumulates significantly more NUOB10-FLAG compared to other transformants with a $p < .01$ (calculated from three biological replicates using two-tailed unequal variances *t*-test).

Primer Name	Sequence (5' to 3')	Target
NUO5 E1L	AGCTAGCACCTCGTCTC	*NUO5*
NUO5 E2L	ACTACAAGCAGAGCGCCATC	
NUO5 E2R	GCTTAGCCACACGGTTC	
PmlI MKS /E2L2	CCGCACGAAAATTGGCAAGTACC	
NUO5 E3R	CATCTCGCCCAGTGTGAAC	
NUO5 E4L	CAACATCCTGGACACCATCAAG	
NUO5 E5R	GTTCAGGTCACGGCAGTAGG	
NUOB10 E1L	CGTCAAAGCTTCCACGATAAGG	*NUOB10*
NUOB10 E2L	CTGAGGGAGCAGGCTCTG	
NUOB10 I2R	CAGAGCCTGCTCCCTCAG	
PDSW-2R	TCATGCTTGCCCGAGAAG	
PDSW-9F	GTCATGAAGCGCCTGCAGG	
NUOB10 E4L	GGCTTCATCTACCACAACAACAG	
NUOB10 E4R	GAAAGGGTTCTCGATCTC	
NUOB10 I3F	CAGTTGGGGTGGGTCCTTC	
PDSW-6R	ATCGCACATGACGGCAG	
PDSW-12R	GAGGAGAGCAGTGGCATCGTG	
PH3	GGAGTCCCGGGATGGATTAAGG	9A2 Cosmid
9A2 PH3	GCCAGGCAACCGCGTCGAG	
APH7 F8	ACTGCTCGCCTTCACCTTC	iHyg3
APH7F	TCGATATCAAGCTTCTTTCTTGC	
APH7R	AAGCTTCCATGGGATGACG	
APH7 R3	AGAATTCCTGGTCGTTCCGCAG	
APH7 R4	TAGGAATCATCCGAATCAATACG	
APH7 R5	CGGTCGAGAAGTAACAGGG	
APH8R5	CACTCACAACCGGGATACC	iPm
AD1	NTCASTWTSGWGTT	Partially degenerate primer
CBLP-F	GCCACACCGAGTGGGTGTCGTGCG	*CBLP*
CBLP-R	CCTTGCCGCCCGAGGCGCACAGCG	
Tub-F	GTCCAAGCTGGGCTTCACCGTC	*TUA2*
Tub-R	GGCGGCAGATGTCGTAGATGGC	
Ubi lower	AGCGTCAGCGGCGGTTGCAGGTATCT	*UBI*
Ubi upper	GTACAGCGGCGGCTACAGGCAC	
EIF1A Fw	CATTGTGGAGCCGCCATTTC	*EIFA*
EIF1A Rev	GGCTGCTTGCATTTGCTTCC	

Supplemental Table S1: Sequence of primers used in this study. The sequence of primers used for TAIL-PCRs, diagnostic PCRs and RT-qPCRs are provided here. *NUO5, NUOB10, CBLP, TUA2, UBI, and EIF1A* are genes in the *Chlamydomonas* genome that correspond to the Phytozome v12 gene numbers

Cre10.g450400, *Cre12.g555150*, *Cre13.g599400* (also referred to as *Cre06.g278222)*, *Cre04.g216850*, *Cre03.g159200*, and *Cre02.g103550*, respectively.

Primer Name	Sequence (5' to 3')	Amplicon
pRS426-NUO5-F	GCGTATTTGACTAGTTCTAGAGCGGCCGCCACCGATTCACGCGTGTACTTG	A (1773 bp)
NUO5E2R	GCTTAGCCACACGGTTC	
NUO5E2L	ACTACAAGCAGAGCGCCATC	B (1532 bp)
NUO5-FLAG-R	GGCCGATTACTTGTCATCGTCGTCCTTATAGTCCGCGGTGGCGTTCAGGTC	
NUO5E2L	ACTACAAGCAGAGCGCCATC	C (1180 bp)
InfuK230R1	CTTGGGCTTGCCTCCCTTACGGATGGTGTCCAGGATGTTGACAATGTCCTTG	
InfuK230RF1	CAAGGACATTGTCAACATCCTGGACACCATCCGTAAGGGAGGCAAGCCCAAG	D (405 bp)
NUO5-FLAG-R	GGCCGATTACTTGTCATCGTCGTCCTTATAGTCCGCGGTGGCGTTCAGGTC	
NUO5-FLAG-F	GACTATAAGGACGACGATGACAAGTAATCGGCCGTGCTCGTG	E (677 bp)
NUO5-pRS426-R2	CAATTAACCCTCACTAAAGGGAACAAAAGCTGGTGATATGACCCAAGCTGCATATG	
pRS426-NUOB10-F	GCGTATTTGACTAGTTCTAGAGCGGCCGCCACCGCTTGCATCGTCCAGGGTAG	F (1734 bp)
C79SRev2	GCGCAGTGCCCGCAGCACGCACGCTCTCCAG	
pRS426-NUOB10-F	GCGTATTTGACTAGTTCTAGAGCGGCCGCCACCGCTTGCATCGTCCAGGGTAG	G (1767 bp)
C91SRev2	GTTGAGTTCCGCACCTCGGTGCTCTTGTGGTAC	
pRS426-NUOB10-F	GCGTATTTGACTAGTTCTAGAGCGGCCGCCACCGCTTGCATCGTCCAGGGTAG	H (1740 bp)
C79SC91SRev	GCTCTTGTGGTACCGCGCAGTGCCCGCAGCACGCACGCTCTCCAG	
pRS426-NUOB10-F	GCGTATTTGACTAGTTCTAGAGCGGCCGCCACCGCTTGCATCGTCCAGGGTAG	I (1780 bp)
NUOB10I2R	ATGTTGAGTTCCGCACCTC	
NUOB10E2L	CTGAGGGAGCAGGCTCTG	J (967 bp)
NUOB10-FLAG-R	CACGCTTTACTTGTCATCGTCGTCCTTATAGTCGTAGCCGCCAGTGGCCTG	
NUOB10-FLAG-F	GACTATAAGGACGACGATGACAAGTAAAGCGTGAGCGCAGCGTG	K (770 bp)
NUOB10-pRS426-R	CAATTAACCCTCACTAAAGGGAACAAAAGCTGGTACTGTAACGAGTACACAG	

C79S Fw<br>NUOB10-FLAG-R	CTGGAGAGCGTGCGTGCTG<br>CACGCTTTACTTGTCATCGTCGTCCTTATAGTCGTAGCCGCCAGTGGCCTG	L (946 bp)
C91S Fw<br>NUOB10-FLAG-R	TACCACAAGTAGCACCGAGGTGCGGAAC<br>CACGCTTTACTTGTCATCGTCGTCCTTATAGTCGTAGCCGCCAGTGGCCTG	M (908 bp)
C79SC91SFw<br>NUOB10-FLAG-R	AGCGTGCGTGCTGCGGGCACTGCGCGGTACCACAAGAGCACCGAG<br>CACGCTTTACTTGTCATCGTCGTCCTTATAGTCGTAGCCGCCAGTGGCCTG	N (946 bp)
APH7F-pRS426<br>APH7R-pRS426	TCGATAAGCTTGATATCGAATTCTCGATATCAAGCTTCTTTCTTG<br>CCCTGGACGATGCAAGCGGTGGCGGCCGCTAAGCTTCCATGGGATGACG	iHyg3 (1796 bp)
APH8F2-pRS426<br>APH8R-pRS426	AAGTACACGCGTGAATCGGTGGCGGCCGCTCAGGCAGACGGGCAGGTG<br>TCGATAAGCTTGATATCGAATTCCCTGGGTACCCGCTTCAAATAC	iPm (1902 bp)

Supplemental Table S2. Primers used for recombination-based cloning in yeast.

The sequence of primers used to amplify the overlapping DNA fragments by PCR is provided here. The nucleotide sequence encoding the FLAG-tag is in red. The site-directed mutations introduced into the primers are indicated with an underline. Each amplicon is given an alphabetical label for easy reference and its size is also provided in the last column.

The amplicons A to N were generated using *Chlamydomonas* genomic DNA (WT, 4C^{-}) as template. The iHyg3 and iPm amplicons were amplified using plasmids pHyg3 and pLS18 as templates, respectively (Berthold *et al.*, 2002, Depege *et al.*, 2003).

Clone Name	Fragments used for recombinant assembly
***In vivo* recombination in yeast**	
pRS426-ble-NUO5-WT-FLAG	pB-NA + A + B + E
pRS426-ble-NUO5-K230R-FLAG	pB-NA + A + C + D + E
pRS426-ble-NUOB10-WT-FLAG	pB-NA + I + J + K
pRS426-ble-NUOB10-C79S-FLAG	pB-NA + F + L + K
pRS426-ble-NUOB10-C91S-FLAG	pB-NA + G + M + K
pRS426-ble-NUOB10-C79SC91S-FLAG	pB-NA + H + N + K
**In-Fusion HD cloning**	
pRS426-iPm-NUO5-WT-FLAG	pRS426-ble-NUO5-WT-FLAG *Eco*RI, *Not*I (9 kb) + iPm
pRS426-iPm-NUO5-K230R-FLAG	pRS426-ble-NUO5-K230R-FLAG *Eco*RI, *Not*I (9 kb) + iPm
pRS426-iHyg3-NUOB10-WT-FLAG	pRS426-ble-NUOB10-WT-FLAG *Eco*RI, *Not*I (9 kb) + iHyg3
pRS426-iHyg3-NUOB10-C79S-FLAG	pRS426-ble-NUOB10-C79S-FLAG *Eco*RI, *Not*I (9 kb) + iHyg3
pRS426-iHyg3-NUOB10-C91S-FLAG	pRS426-ble-NUOB10-C91S-FLAG *Eco*RI, *Not*I (9 kb) + iHyg3
pRS426-iHyg3-NUOB10-C79SC91S-FLAG	pRS426-ble-NUOB10-C79SC91S-FLAG *Eco*RI, *Not*I (9 kb) + iHyg3

Supplemental Table S3. Method of plasmid construction.

The PCR fragments described in Table S2 carry overlapping segments that will enable recombinant cloning. The fragments used for generating each clone are detailed here. pB-NA refers to the vector pRS426-ble vector linearized (6864 bp) with *Not*I *and Ale*I restriction enzymes. The *NUO5* and *NUOB10* genes were cloned into the pRS426-ble vector by *in vivo* recombination in yeast. Subsequently, the *ble* selection marker was substituted by *in vitro* recombination using the In-Fusion HD enzyme.

Transformant name	Recipient strain	Plasmid used for biolistic transformation	Selection marker	Selection medium
amc9; *NUO5* [CC-5602]	*amc9 (41D9) strain* (*mt*$^{-}$; *nuo5::APHVII*; *arg7-8)* [CC-5601]	Cosmid 9A2 (contains wild-type *NUO5* gene, no tag)	*ARG7*	TAP
amc9; *NUO5-FLAG* [CC-5603]		pRS426-iPm-NUO5-WT-FLAG	*APHVIII*	TARG + Pm
amc9; *NUO5*^{K230R}*-FLAG* [CC-5604]		pRS426-iPm-NUO5-K230R-FLAG	*APHVIII*	TARG + Pm
amc5; *NUOB10* [CC-5592]	*amc5 (87D3)* strain (*mt*$^{+}$; *nuob10::APHVIII*; *arg7-8*) [CC-5591]	Cosmid 7D10 (contains wild-type NUOB10 gene, no tag)	*ARG7*	TAP
amc5; *NUOB10-FLAG* [CC-5593]		pRS426-iHyg3-NUOB10-WT-FLAG	*APHVII*	TARG + HyB
amc5; *NUOB10*^{C79S}*-FLAG* [CC-5594]		pRS426-iHyg3-NUOB10-C79S-FLAG	*APHVII*	TARG + HyB
amc5; *NUOB10*^{C91S}*-FLAG* [CC-5595]		pRS426-iHyg3-NUOB10-C91S-FLAG	*APHVII*	TARG + HyB
amc5; *NUOB10*$^{C79S\ C91S}$*-FLAG* [CC-5596]		pRS426-iHyg3-NUOB10-C79SC91S-FLAG	*APHVII*	TARG + HyB

Supplemental Table S4. Summary of biolistic transformation.

The *amc9* and *amc5* recipient strains were plated on the specified selective medium (column five) and bombarded with tungsten particles coated with the respective plasmids. Resulting transformants were screened by diagnostic PCR for the presence of the transgene and the

site-directed mutations were confirmed by sequencing. The transformant chosen for further analysis is indicated in column one. The recipient strain used for generating each transformant is provided in column two. The plasmids used to generate the transformants and the respective selection markers are detailed in columns three and four, respectively. The Chlamydomonas Resource Center reference numbers for the strains are provided in square brackets.

REFERENCES

Banroques, J., Delahodde, A., and Jacq, C. (1986) A mitochondrial RNA maturase gene transferred to the yeast nucleus can control mitochondrial mRNA splicing. *Cell* **46**: 837-844.

Barbieri, M.R., Larosa, V., Nouet, C., Subrahmanian, N., Remacle, C., and Hamel, P.P. (2011) A forward genetic screen identifies mutants deficient for mitochondrial complex I assembly in *Chlamydomonas reinhardtii*. *Genetics* **188**: 349-358.

Berthold, P., Schmitt, R., and Mages, W. (2002) An engineered *Streptomyces hygroscopicus aph 7*" gene mediates dominant resistance against hygromycin B in *Chlamydomonas reinhardtii*. *Protist* **153**: 401-412.

Birrell, J.A., Morina, K., Bridges, H.R., Friedrich, T., and Hirst, J. (2013) Investigating the function of [2Fe-2S] cluster N1a, the off-pathway cluster in complex I, by manipulating its reduction potential. *Biochem J* **456**: 139-146.

Chen, D.C., Yang, B.C., and Kuo, T.T. (1992) One-step transformation of yeast in stationary phase. *Curr Genet* **21**: 83-84.

de Montaigu, A., Sanz-Luque, E., Macias, M.I., Galvan, A., and Fernandez, E. (2011) Transcriptional regulation of CDP1 and CYG56 is required for proper NH4+ sensing in Chlamydomonas. *J Exp Bot* **62**: 1425-1437.

Depege, N., Bellafiore, S., and Rochaix, J.D. (2003) Role of chloroplast protein kinase Stt7 in LHCII phosphorylation and state transition in Chlamydomonas. *Science* **299**: 1572-1575.

Dreyfuss, B.W., Hamel, P.P., Nakamoto, S.S., and Merchant, S. (2003) Functional analysis of a divergent system II protein, Ccs1, involved in *c*-type cytochrome biogenesis. *J .Biol. Chem.* **278**: 2604-2613.

Hall, T.A. (1999) BioEdit: a user-friendly biological sequence alignment editor and analysis program for Windows 95/98/NT. *Nucleic Aciids Symposium Series* **41**: 95-98.

Harris, E.H., (1989) *The Chlamydomonas Sourcebook: A comprehensive guide to biology and laboratory use*. Academic Press, San Diego, CA.

Kropat, J., Hong-Hermesdorf, A., Casero, D., Ent, P., Castruita, M., Pellegrini, M., *et al.* (2011) A revised mineral nutrient supplement increases biomass and growth rate in Chlamydomonas reinhardtii. *Plant J* **66**: 770-780.

Livak, K.J., and Schmittgen, T.D. (2001) Analysis of relative gene expression data using real-time quantitative PCR and the 2(-Delta Delta C(T)) Method. *Methods* **25**: 402-408.

Newman, S.M., Boynton, J.E., Gillham, N.W., Randolph-Anderson, B.L., Johnson, A.M., and Harris, E.H. (1990) Transformation of chloroplast ribosomal RNA genes in Chlamydomonas: molecular and genetic characterization of integration events. *Genetics* **126**: 875-888.

Nishioka, K., Vilarino-Guell, C., Cobb, S.A., Kachergus, J.M., Ross, O.A., Hentati, E., *et al.* (2010) Genetic variation of the mitochondrial complex I subunit NDUFV2 and Parkinson's disease. *Parkinsonism Relat Disord* **16**: 686-687.

Noor-Mohammadi, S., Pourmir, A., and Johannes, T.W. (2014) Method for assembling and expressing multiple genes in the nucleus of microalgae. *Biotechnol Lett* **36**: 561-566.

Saint-Georges, Y., Bonnefoy, N., di Rago, J.P., Chiron, S., and Dujardin, G. (2002) A pathogenic cytochrome b mutation reveals new interactions between subunits of the mitochondrial bc1 complex. *J Biol Chem* **277**: 49397-49402.

Sambrook, J., Fritsch, E.F., and Maniatis, T., (1989) *Molecular Cloning A Laboratory Manual*. Cold Spring Harbor Laboratory Press, New York.

Sazanov, L.A., and Hinchliffe, P. (2006) Structure of the hydrophilic domain of respiratory complex I from *Thermus thermophilus*. *Science* **311**: 1430-1436.

Schneider, C.A., Rasband, W.S., and Eliceiri, K.W. (2012) NIH Image to ImageJ: 25 years of image analysis. *Nat Methods* **9**: 671-675.

Shimogawara, K., Fujiwara, S., Grossman, A., and Usuda, H. (1998) High-efficiency transformation of *Chlamydomonas reinhardtii* by electroporation. *Genetics* **148**: 1821-1828.

Sievers, F., Wilm, A., Dineen, D., Gibson, T.J., Karplus, K., Li, W., *et al.* (2011) Fast, scalable generation of high-quality protein multiple sequence alignments using Clustal Omega. *Mol Syst Biol* **7**: 539.

Verkhovskaya, M.L., Belevich, N., Euro, L., Wikstrom, M., and Verkhovsky, M.I. (2008) Real-time electron transfer in respiratory complex I. *Proc Natl Acad Sci U S A* **105**: 3763-3767.

Werner, R., and Mergenhagen, D. (1998) Mating Type Determination of *Chlamydomonas reinhardtii* by PCR. *Plant Mol. Biol. Rep,* **16**: 295-299.
