# Supplementary material for: Chlamydomonas reinhardtii as a plant model system to study mitochondrial complex I dysfunction
Source: Plant Direct. 2020 Feb 3;4(2):e00200. doi: 10.1002/pld3.200 (PMC6996877; doi:10.1002/pld3.200)
Supplement: Supplementary file 2 [file PLD3-4-e00200-s002.pdf]

December 19, 2019

Dear Editor,

We thank you for your consideration of our manuscript entitled "*Chlamydomonas reinhardtii* as a plant model system to study mitochondrial complex I dysfunction" for publication in *Plant Direct.* As per your recommendation, we are now submitting a revised version of the manuscript in accordance with the comments received from the reviewers and the editor. We sincerely thank the reviewers for their valuable suggestions for improving the manuscript and have incorporated all the suggestions we deemed relevant in the revised version. In addition, we have addressed the reviewers' comments below. We hope that you will find this revised version of our manuscript is improved and acceptable for publication in your journal.

Reviewer #1

1) I suggest just one change, move the Figure 6A to the Supplemental figures; the primers are only really visually explained there and seeing that in Figure 6 isn't required to understand the experiment. I also think B could go as well, then perhaps Figure 6C could be combined with 7A and B.

   We have incorporated the requested change.

2) One point I'd like to emphasize is that the pileup comparisons of the NUOB5's shows that the mutated Lys230 to Arg in humans associated with Parkinson's is Arg in Arabidopsis and grapes, a hallmark of a residue that must be positively charged, but not have the Lys identity. One might have guessed from just doing the pileup that no phenotype would result, but this test in Chlamy was suggestive that the linkage likely is not telling in humans.

   We agree with the reviewer's observation and have included a statement in lines 539-541. While there was the possibility that the conservative substitution might not elicit a negative impact on complex I function, there have been instances where even a lysine-to-arginine substitution in enzymes have resulted in 500 times slower activity (Morris *et al.*, 1996). In this study, we have experimentally shown that in the case of NUO5 Lys230, this substitution does not appear to affect complex I activity or assembly.

Reviewer #2

1) A limitation in the discussion is that the observation in Chlamydomonas is not always true in human mitochondria. Some assembly machineries may be specific to organisms. A good example is the impact of the K209R mutation reported in this manuscript. The present discussion sounds too strong.
   We acknowledge this limitation and we have added a couple of sentences in lines 542-547 and 629-632.

2) Line 151. 50 µmol m-2 s-1 is low light.

In our laboratory conditions, 50 µmol m^{-2} s^{-1} is high light, where as our low light conditions are 0.5-1 µmol m^{-2} s^{-1}. Keeping in mind, that other laboratories might define high light and low light differently according to the capacity of their incubators, we have added a definition for high light and low light in Lines 156-157.

3) Lines 498-499. defective for photosystem I cyclic electron transfer that in involved in ATP production in chloroplasts...

The requested change has been incorporated (Line 516)

4) Lines 883-885 and corresponding positions of other legends. The symbol (#) indicates the chlorophyll-containing complexes...

The statement has now been uniformly incorporated in all figure legends.

To be uniform with recent updates to the nomenclature in the field of Chlamydomonas, the following additional changes have been made:

- *aph7"* and *aph8"* transgenes are now changed to *APHVII* and *APHVIII*. These changes have been made to the main text, supplemental text, Supplemental Figures S4, S8.
- The genotypes for *Chlamydomonas* strains are separated by a semi-colon instead of a comma.
- The strains transformed with transgenes are represented differently. For example, *amc9* (*NUO5)* transformant is now referred to as [*amc9*; *NUO5*]. These changes have been applied to Figures 3-6 and supplemental figures S4, S6, S8, and S9.
- In the bar graphs of Figures 1, 4, and 6, the previous version mistakenly displayed standard error instead of standard deviation. Corrections have been made to the error bars to reflect standard deviation as previously described in the figure legends. This correction does not change the mean or *p*-values in any of the data.

Sincerely,

Patrice Hamel, Ph.D.
Associate Professor
Department of Molecular Genetics
Department of Molecular and Cellular Biochemistry
582 Aronoff laboratory, 318 W. 12th Avenue
Columbus, OH 43210
Tel:(614)-292-3817 **Fax**:(614)-292-6345

Bibliography

Morris AJ, Davenport RC, Tolan DR. 1996. A lysine to arginine substitution at position 146 of rabbit aldolase A changes the rate-determining step to Schiff base formation. Protein Eng **9**, 61-67.
